# Stochastic combinations of actin regulatory proteins are sufficient to drive filopodia formation

**DOI:** 10.1083/jcb.202003052

**Published:** 2021-03-18

**Authors:** Ulrich Dobramysl, Iris Katharina Jarsch, Yoshiko Inoue, Hanae Shimo, Benjamin Richier, Jonathan R. Gadsby, Julia Mason, Alicja Szałapak, Pantelis Savvas Ioannou, Guilherme Pereira Correia, Astrid Walrant, Richard Butler, Edouard Hannezo, Benjamin D. Simons, Jennifer L. Gallop

**Affiliations:** 1 Gurdon Institute, University of Cambridge, Cambridge, UK; 2 Department of Biochemistry, University of Cambridge, Cambridge, UK; 3 Institute of Science and Technology Austria, Klosterneuburg, Austria; 4 Department of Applied Mathematics and Theoretical Physics, University of Cambridge, Cambridge, UK

## Abstract

Assemblies of actin and its regulators underlie the dynamic morphology of all eukaryotic cells. To understand how actin regulatory proteins work together to generate actin-rich structures such as filopodia, we analyzed the localization of diverse actin regulators within filopodia in *Drosophila *embryos and in a complementary in vitro system of filopodia-like structures (FLSs). We found that the composition of the regulatory protein complex where actin is incorporated (the filopodial tip complex) is remarkably heterogeneous both in vivo and in vitro. Our data reveal that different pairs of proteins correlate with each other and with actin bundle length, suggesting the presence of functional subcomplexes. This is consistent with a theoretical framework where three or more redundant subcomplexes join the tip complex stochastically, with any two being sufficient to drive filopodia formation. We provide an explanation for the observed heterogeneity and suggest that a mechanism based on multiple components allows stereotypical filopodial dynamics to arise from diverse upstream signaling pathways.

## Teaser

Dobramysl et al. find that protein complexes at filopodial tips are strongly heterogeneous. Using a combination of in vivo imaging in *Drosophila*, a cell-free system, and mathematical modeling, they propose that pairs of proteins form distinct subcomplexes that integrate diverse regulatory pathways to drive constrained filopodial dynamics.

## Introduction

The regulation of actin polymerization is crucial for numerous cell functions, including cell migration, adhesion, and epithelial closure (Jacinto et al., 2002; Jacquemet et al., 2015) and is often disrupted in disease, such as cancer metastasis and intracellular infection by pathogens (Bendris and Schmid, 2017; Molinie and Gautreau, 2018; Stradal and Schelhaas, 2018; Tan et al., 2013). Micron-scale actin superstructures and their associated regulators form transient membrane-bound complexes that orchestrate large-scale cytoskeletal remodeling and provide the mechanical infrastructure for the cell (Lappalainen, 2016; Senju et al., 2017). One of the best examples is filopodia, with their characteristic membrane-associated “tip complex” where new actin monomers are incorporated, leading to rapid extension of the filopodia from the cell surface (Applewhite et al., 2007; Mallavarapu and Mitchison, 1999). The tip complex contains many components, including formins such as diaphanous-related formin 3 (Diaph3), barbed-end polymerases Enabled (Ena), vasodilator-stimulated phosphoprotein (VASP), actin bundling proteins including Fascin, and the molecular motor myosin X. There are currently three main models for filopodia formation, each identifying specific tip complex proteins as the key players: (1) formins mediating de novo actin nucleation (Block et al., 2008; Faix et al., 2009; Steffen et al., 2006); (2) a preexisting actin network generated by the Arp2/3 complex becoming bundled by Fascin (Svitkina et al., 2003; Vignjevic et al., 2003; Yang and Svitkina, 2011); and (3) membrane-bound adaptor proteins recruiting Ena/VASP (Disanza et al., 2013; Mattila et al., 2007), which could coexist with either formin or Arp2/3 complex–based mechanisms. One way to reconcile these models is to postulate the existence of subtypes of filopodia on the basis of their mechanism of formation (Barzik et al., 2014; Bilancia et al., 2014; Mellor, 2010; Nowotarski et al., 2014; Young et al., 2015). What is not yet clear is whether the subtypes reflect differences between cell types or coexist in the same cell and whether they impart particular properties to the growing filopodia. We recently examined this question by measuring whether the amount of Ena and VASP at the tip complex correlated with the protrusion velocity of filopodia, using cultured *Xenopus* retinal ganglion cells (Urbančič et al., 2017). We observed a correlation in only a subset of filopodia, suggesting that the accumulation of Ena/VASP proteins is not essential and there are diverse molecular mechanisms that lead to filopodial elongation.

Here, we comprehensively analyze the role of heterogeneity in the filopodial tip complex. By measuring endogenously tagged actin regulators in *Drosophila,* we confirmed similar heterogeneity to exogenous expression in *Xenopus* retinal ganglion cells. We found that a cell-free system of filopodia-like structures (FLSs) is characterized by similar heterogeneities, and it allowed us to make large-scale combinatorial measurements of the correlations of actin regulators with each other and the morphology of the actin bundle. We discovered that the emergence of FLSs and their resulting lengths are remarkably insensitive to the presence or absence of any individual tip complex protein. By measuring the momentary rates of growth and shrinkage of the actin bundle and incorporating theoretical modeling, we identified a simple theory that suggests a mechanistic role for tip complex heterogeneity, and we tested its predictions in vitro and in vivo. Our work explains how diverse combinations of tip complex proteins give rise to filopodia.

## Results

### Heterogeneous tip complexes and exponentially distributed filopodial lengths in vivo in *Drosophila*

**Figure 1.**
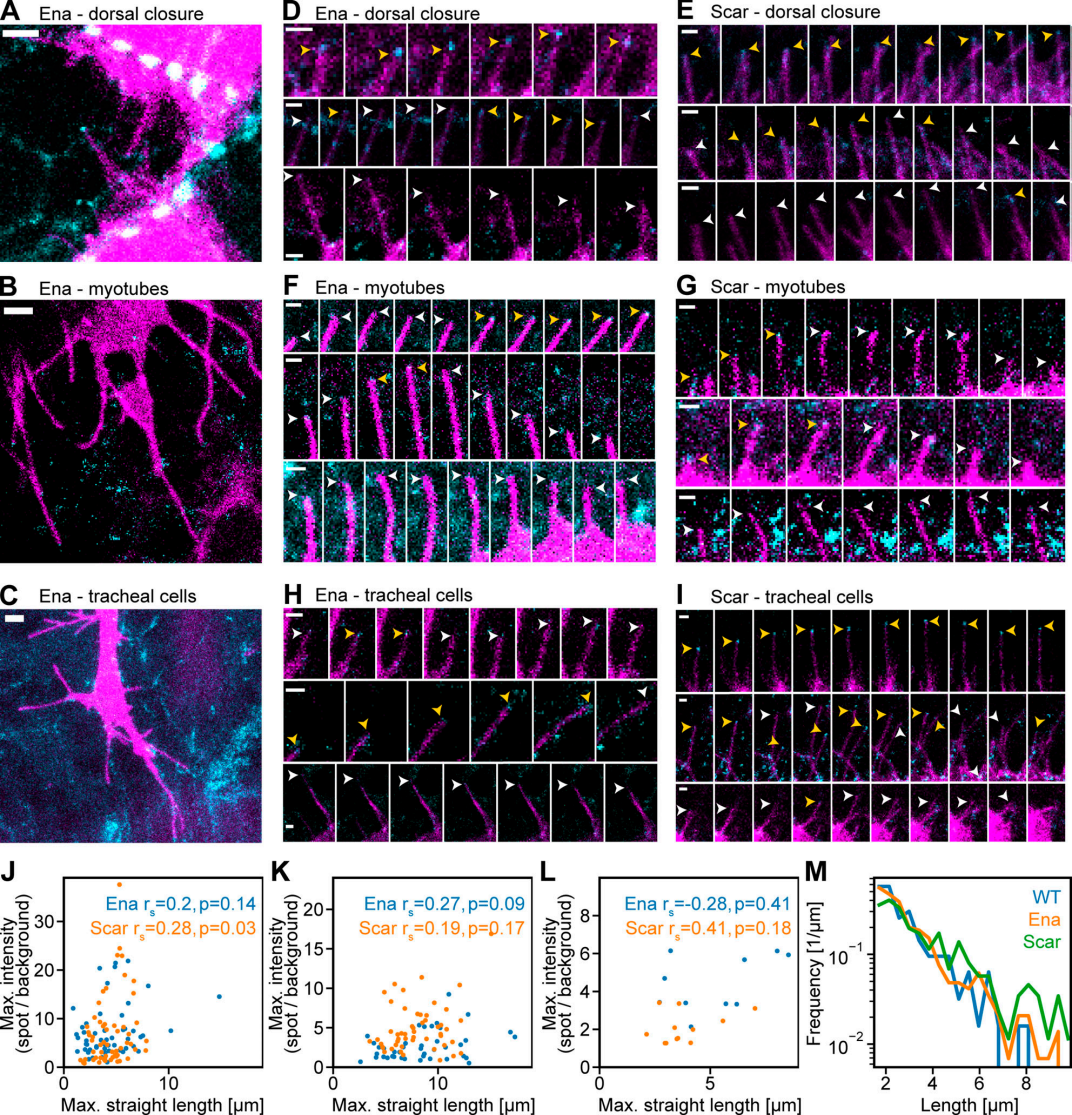
**Heterogeneity in Ena and Scar at filopodium tips accompanies exponentially distributed filopodium lengths in vivo.** **(A–C)** Varying intensities of Ena (cyan) at the tips of filopodia (membrane marker expressed using the Gal4 system shown in magenta) in leading edge cells in dorsal closure (A), myotubes (B), and tracheal cells (C) in the *Drosophila* embryo. Pictures are maximum-intensity projections of the cell marker together with the filopodium tip z-slice of the Ena channel. Scale bars = 2 μm. **(D)** Time-lapse montages every 15 s of filopodia in leading edge cells in dorsal closure in the *Drosophila* embryo with fluorescent Ena. Yellow and white arrowheads indicate filopodium tips with and without protein, respectively. Scale bar = 1 µm. **(E–H)** Similarly, Scar in dorsal closure (E), Ena in myotubes (F), Scar in myotubes (G), Ena in tracheal cells (H), and Scar in tracheal cells (I). **(J–L)** Scatter plot of maximal (Max.) Ena and Scar intensities at filopodium tips versus maximal end-to-end (straight) filopodia length, respectively, in dorsal closure (*n* = 53 and 58), tracheal cells (*n* = 42 and 51), and myotubes (*n* = 10 and 12). Spearman correlation coefficients and associated P values are shown. **(M)** Log plots of filopodium lengths from control (*n* = 150), GFP-Ena (*n* = 343), and mNeonGreen-Scar/WAVE (*n* = 213) knock-in homozygous flies are exponentially distributed and largely similar.

We first examined the localization of Ena and the Arp2/3 complex nucleation promoting factor Scar/WAVE at the filopodial tip complex in vivo using the *Drosophila* embryo. Ena is known to contribute to filopodial extension while Scar/WAVE was previously localized to filopodia tips in *Drosophila* by overexpression (Hahne et al., 2001; Nozumi et al., 2003), and reducing its levels reduces filopodia in *Drosophila* BG2 cells (Biyasheva et al., 2004). We used *Drosophila* to give a native mechanical and signaling environment and reduce any potential redundancy due to paralogs. To ensure normal levels of expression, we used CRISPR/Cas9 genome editing of endogenous loci to tag the proteins with fluorescent proteins (GFP and mNeonGreen). Whereas loss-of-function mutations at these two loci cause severe phenotypes (Gertler et al., 1990; Zallen et al., 2002), flies homozygous for the tagged genes did not display any defects in viability, appearance, or fertility, indicating that the fluorescent protein fusion does not substantially impair function. We performed high-resolution time-lapse imaging at filopodia tips in leading-edge epidermal cells during dorsal closure, at the tips of lateral transverse myotubes as they target their attachment sites, and in tracheal cells as they extend into the embryo (Fig. 1, A–C: and Video 1, Video 2, Video 3, Video 4, Video 5, and Video 6). Each cell type was marked by the expression of the fluorescent membrane marker CD8-mCherry to distinguish filopodia from the surrounding tissues (Fig. 1, magenta). Considerable heterogeneity in Scar and Ena intensity at filopodia tips was evident in each of the tissues (Fig. 1, D–I). Each tissue had some filopodia with a strong spot of Ena or Scar at the tip that tracks the filopodium (Fig. 1, D–I, top row). There were instances where a spot was present but was less bright or was present for shorter times (Fig. 1, D–I, middle row) and instances where there was no spot detectable (Fig. 1, D–I, bottom row). This confirmed the heterogeneity that we previously observed by exogenous expression of Ena and VASP in *Xenopus* neuronal filopodia (Urbančič et al., 2017) and is consistent with the finding that filopodia numbers and length are only reduced by ∼60% on sequestration of Ena to mitochondria or in *ena* maternal/zygotic *Drosophila* mutants (Gates et al., 2007), demonstrating that it is still possible to make filopodia without Ena. Similarly, cells deficient in Scar and Arp2/3 complex also still support filopodial projections (Kunda et al., 2003; Steffen et al., 2006; Steffen et al., 2004). The filopodia with differing tip complex accumulation of Ena and Scar can be adjacent within the same cells, suggesting that different subtypes of filopodia arise from the same intracellular and extracellular environments. Quantification of the images confirmed that there was little correlation between the final maximal length of the filopodia and the maximal intensity of Ena or Scar fluorescence at the filopodial tip (Fig. 1, J–L).

**Video 1.** **Maximum-intensity projection of laser scanning confocal time-lapse z-stacks of *enaGFP, en-Gal4;UAS-cd8mCherry* embryos, late stage 14.** Representative video of a total of 12 videos from seven embryos. Scale bar represents 10 µm. Images were captured at 15-s intervals and are replayed at 10 frames per second. Ena is in cyan, membrane in magenta.

**Video 2.** **Maximum-intensity projection of laser scanning confocal time-lapse z-stacks of *Scar/WAVENeonGreen en-Gal4;UAS-cd8mCherry* embryos, late stage 14. **Representative video of a total of 13 videos from eight embryos. Scale bar represents 10 µm. Images were captured at 15-s intervals and are replayed at 10 frames per second. Scar/WAVE is in cyan, membrane in magenta.

**Video 3.** **Maximum-intensity projection of laser scanning confocal time-lapse z-stacks of *enaGFP, btl-Gal4;UAS-cd8mCherry* embryos, late stage 14. **Representative video of of a total of 24 videos from 20 embryos. Scale bar represents 10 µm. Images were captured at 15-s intervals and are replayed at 10 frames per second. Ena is in cyan, membrane in magenta.

**Video 4.** **Maximum-intensity projection of laser scanning confocal time-lapse z-stacks of *Scar/WAVENeonGreen;btl-Gal4 UAS-CAAXCherry* embryos, stage 15. **Representative video of a total of nine videos from six embryos. Scale bar represents 10 µm. Images were captured at 15-s intervals and are replayed at 10 frames per second. Scar/WAVE is in cyan, membrane in magenta.

**Video 5.** **Maximum-intensity projection of laser scanning confocal time-lapse z-stacks of *enaGFP, mef2-Gal4 UAS-CAAXCherry* embryos, stage 15. **Representative video of a total of 15 videos from 12 embryos. Scale bar represents 10 µm. Images were captured at 15-s intervals and are replayed at 10 frames per second. Ena is in cyan, membrane in magenta.

**Video 6.** **Maximum-intensity projection of laser scanning confocal time-lapse z-stacks of *Scar/WAVENeonGreen;mef2-Gal4 UAS-CAAXCherry* embryos, stage 15. **Representative video of a total of 12 videos from eight embryos. Scale bar represents 10 µm. Images were captured at 15-s intervals and are replayed at 10 frames per second. Scar/WAVE is in cyan, membrane in magenta.

We asked whether there were subtypes of filopodia characterized by their final length by measuring large numbers of leading edge filopodia during dorsal closure. Similar to our previous measurements in myotube filopodia (Richier et al., 2018), we observed a remarkably exponential-like distribution of filopodial lengths (Fig. 1 M, which is also observed with tagged Ena and Scar, verifying their utility). Such a distribution provides evidence for an underlying mechanism based on stochasticity of filopodial growth and shrinkage rather than subtypes of filopodia, as in that case we would expect to see a multimodal or more complex distribution.

### The assembly of FLSs shows similar properties as native filopodia

To analyze the contributions of more actin regulatory proteins at higher time resolution and with a more complete set of morphological parameters of the actin bundle than is possible in cells, we asked whether our cell-free FLS system shared similar properties with native filopodia (Lee et al., 2010). This system uses *Xenopus* egg extracts and a phosphatidylinositol (4,5)-bisphosphate–supported lipid bilayer to reconstitute the growth of long actin bundles from membrane-localized complexes of actin regulatory proteins that resemble the filopodial tip complex (which we denote as the tip complex by analogy). FLSs are not a strict filopodia mimic as membrane does not surround the shaft, offering less spatial constraint to tip complex protein accumulation than is the case in filopodia, and neural–Wiskott Aldrich syndrome protein (N-WASP) is used as an Arp2/3 complex activator rather than the closely related Scar/WAVE protein that seems to fulfill a similar role in *Drosophila*. Nevertheless, FLSs contain actin filaments bundled by Fascin, and new actin monomers are incorporated at complexes that include Ena, VASP, and diaphanous-related formin 3 (Diaph3; Lee et al., 2010).

**Figure 2.**
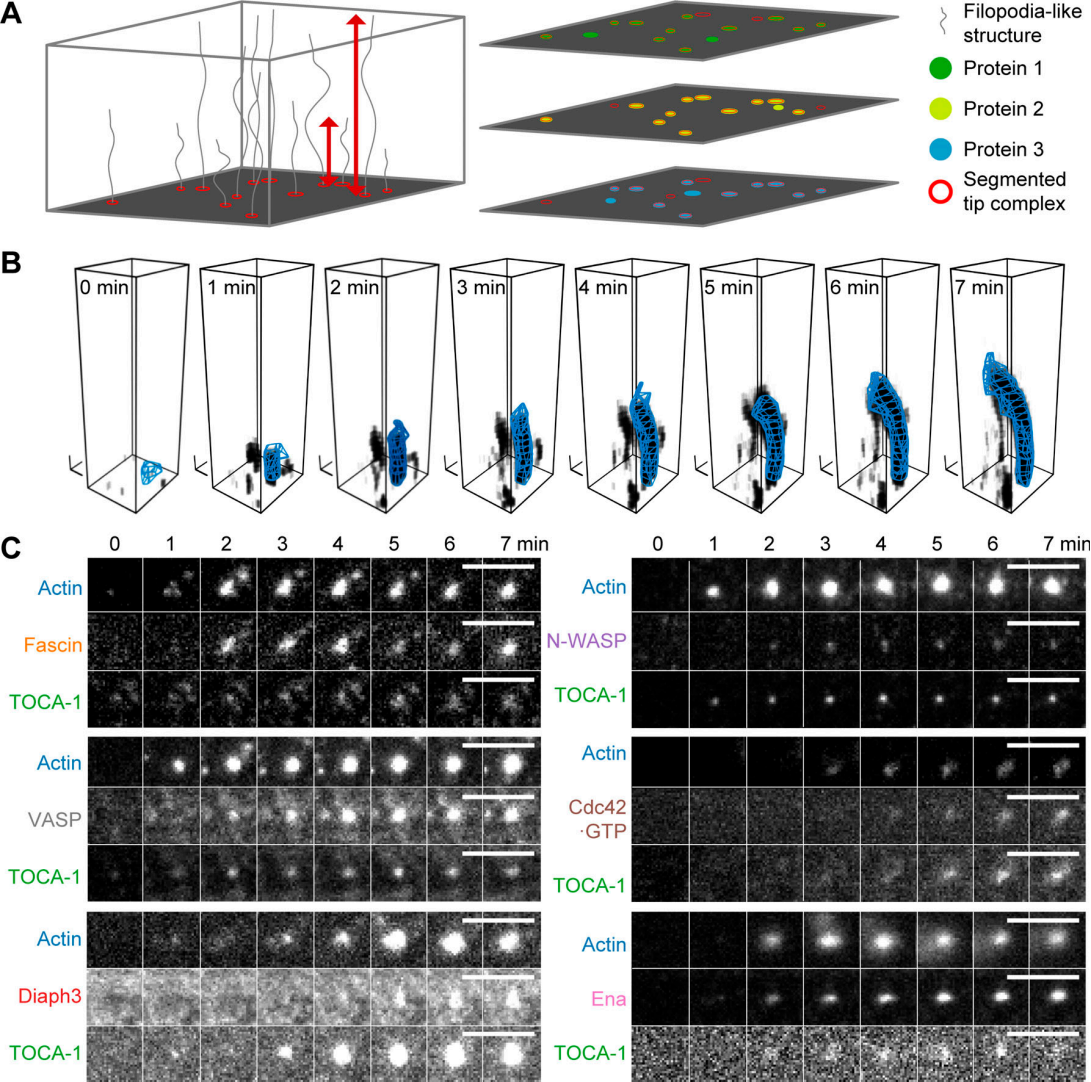
**FLS growth and segmentation of actin bundles and tip complex assembly.** **(A)** Images from HILO and confocal or wide-field illumination on the same fields of view. Individual FLSs are segmented based on the actin fluorescence from the z-stack. Red arrows indicate straight tip-to-end distance. A mask on the actin channel is used as an overlay on any other channel, measuring intensities inside the tip complex area defined at the membrane with background correction (at the base slice), and along the shaft. Output includes protein intensity information and shape parameters of the bundle. **(B)** Single example of bundle growth in time, together with its 3D reconstruction (blue mesh). Actin structures are shown by setting each voxel’s transparency according to its measured intensity (darker indicates higher intensity). The 3D reconstruction overlay uses the output of FLS Ace rendered using Blender software. Black scale tripods indicate 1 μm along each axis. **(C)** Time-lapse montages of six example FLS tip complexes showing the intensity increase of actin, TOCA-1, and another tip complex protein (scale bars = 5 µm). Protein concentrations labeled/unlabeled were Actin 210/14,000; TOCA-1 10/3; VASP 20/16; N-WASP 20/1; GBD 2/0; Fascin 300/416; Diaph3: 20/10; and Ena: 40/40 in nanomolars.

**Figure S1.**
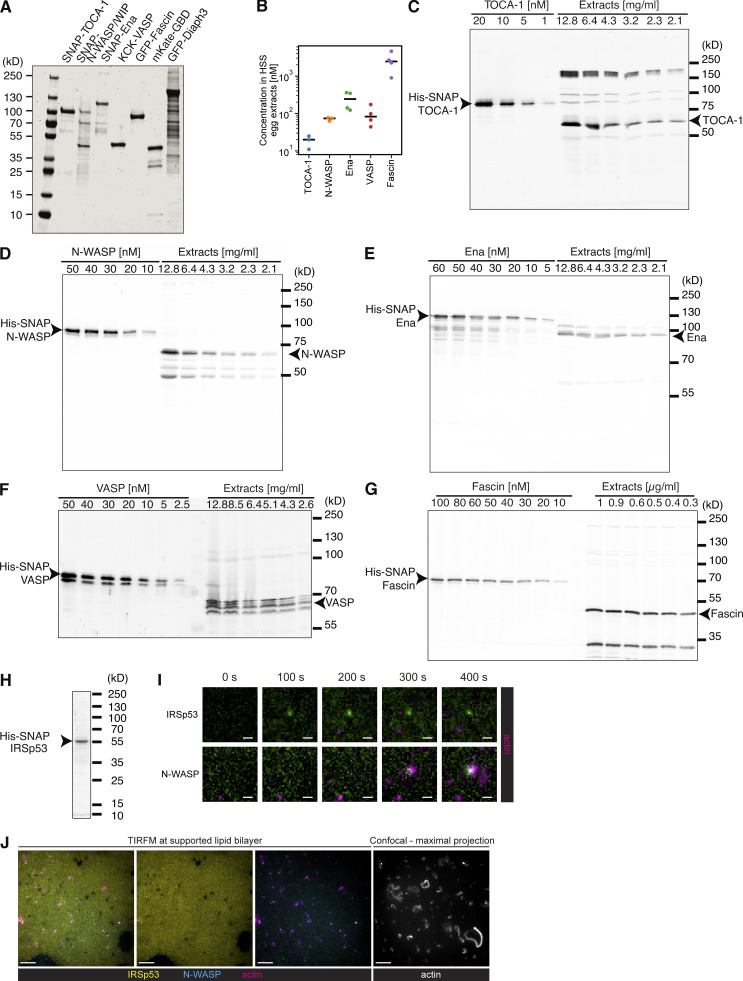
**Purified protein gels and quantitative Western blots.** **(A)** Coomassie staining of purified protein size separated via SDS-PAGE. **(B)** Quantification of three or four independent measurements for endogenous actin regulators within the HSS extracts. Black horizontal lines indicate the measurement mean. **(C–G)** Example blots of purified protein and *X. laevis* egg HSS extracts used to calculate concentration of proteins via quantitative Western blotting, probed as TOCA-1 (C), N-WASP (D), Ena (E), VASP (F; where we combined the bands suspecting they represented different phosphorylation states), and Fascin (G). **(H)** Purified IRSp53. **(I)** Time course showing that Alexa488-SNAP-IRSp53 foci do not localize to FLS actin bundles, whereas control N-WASP does (both added at 50 nM). Scale bars = 1 μm. **(J)** Alexa488-SNAP-IRSp53 binds the supported lipid bilayer. Scale bars = 10 μm. HSS, high-speed supernatant. TIRFM, total internal reflection fluorescence microscopy.

This assay allows tracking and quantification of the FLS tip complexes and actin bundles in large numbers using multichannel fluorescence microscopy (Fig. 2, A and B). To determine how the actin regulatory proteins assemble into tip complexes in vitro, we chose seven proteins representing the major known regulators of actin polymerization in FLSs (Lee et al., 2010): the BAR-SH3 domain containing protein Transducer of cell division cycle 42 (Cdc42) activation-1 (TOCA-1), which is linked to filopodia, together with N-WASP in neuroblastoma cells (Bu et al., 2009; which we included in all experiments as a reference, because it arrives first), together with Cdc42 (where we measured the GTP-bound state using the G protein–binding domain [GBD] of N-WASP), N-WASP, Ena, VASP, Diaph3, or Fascin plus actin (Table 1; Fig. S1 A shows a Coomassie gel of the purified proteins and Fig. S1, B–G shows our determination of protein concentration in the extracts). While I-BAR protein IRSp53 localizes to the supported lipid bilayer, it does not enrich at FLS tip complexes (Fig. S1, H–J), which agrees with the shaft rather than tip localization observed for endogenous IRSp53 in filopodia in B16F1 cells (Cheng and Mullins, 2020). We optimized the signal-to-noise-ratio while keeping within reasonable range of the endogenous protein levels when adding labeled protein and monitored the appearance and accumulation of actin regulators at the FLS tip complex using highly inclined and laminated optical sheet (HILO) illumination at the membrane, with spinning disk confocal microscopy of the same samples in the actin channel in the volume above the coverslip (Fig. 2, B and C; and Table 1).

**Table 1.** Protein combinations used to measure kinetics and steady-state tip complex composition with two regulators

Actin fluorophore	SNAP TOCA-1 fluorophore	Third protein	Number of time-lapse experiments	Number of time-lapse fields of view	Number of snapshot experiments	Number of snapshot fields of view
Alexa 568	Alexa 647	eGFP-Diaph3	3	9	6	10
Alexa 568	Alexa 647	eGFP-Fascin	2	6	5	46
Alexa 568	Alexa 488	SNAP–Ena Alexa 647	3	9	6	43
Alexa 647	Alexa 488	mKate-GBD	3	12	2	26
Alexa 647	Alexa 488	KCK–VASP Alexa 568	2	8	3	16
Alexa 568	Alexa 488	SNAP–N-WASP Alexa 647	5	17	0	0

eGFP, enhanced GFP.

**Figure S2.**
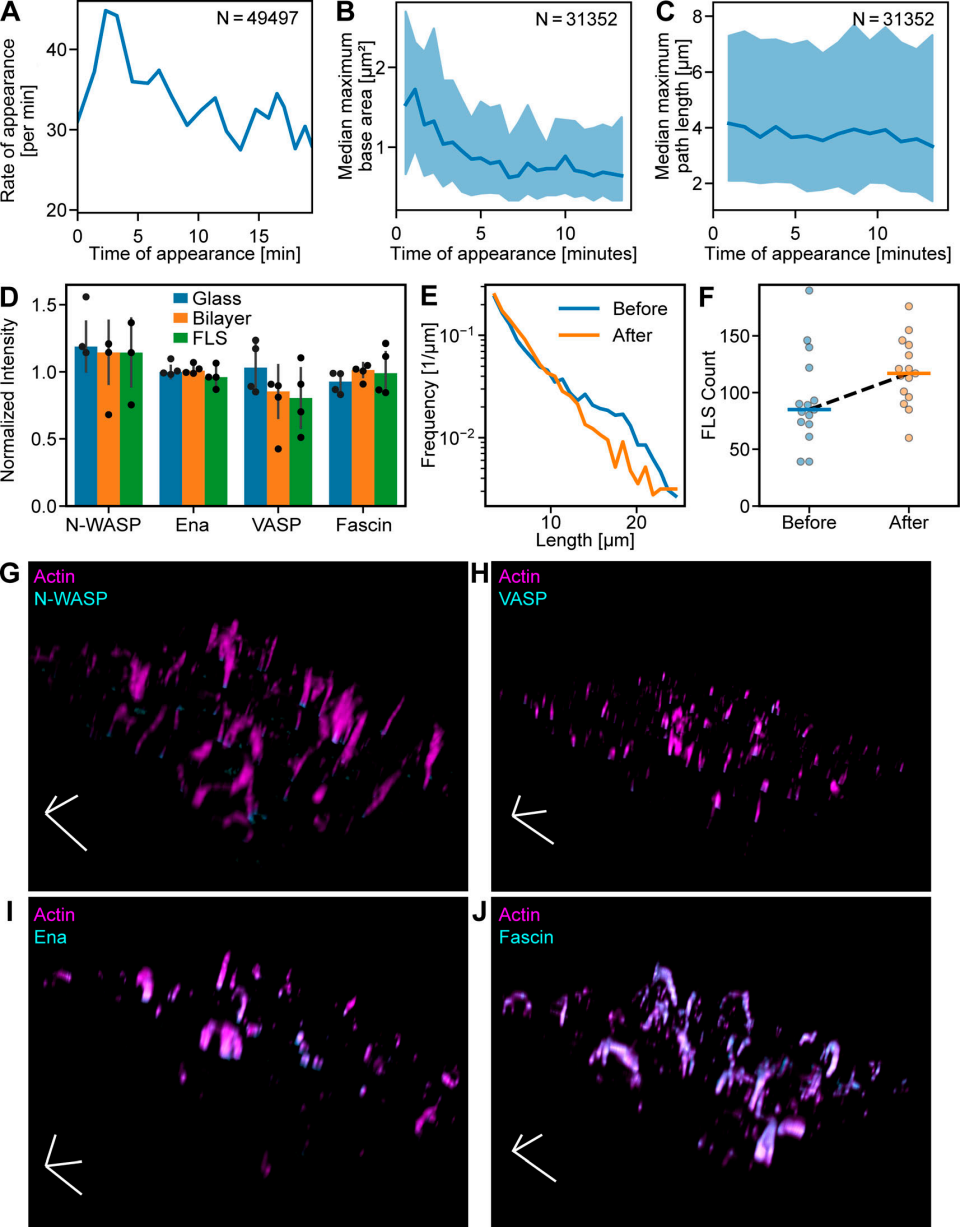
**FLS appearance and morphology over time.** **(A)** Rate of appearance of FLSs with time. **(B)** FLSs that arise early in the experiment end up slightly larger. **(C)** The lengths of FLSs that arise early and late are similar. Shaded areas in all graphs are the standard deviation. **(D)** Western blot quantification of N-WASP, Ena, VASP, and Fascin in FLS assay mix after incubation on coverslip glass surface, supported lipid bilayer, and after an FLS assay was performed for 20 min, normalized to the loading control (fluorescent actin) and to the protein intensity in the preincubation mix (*n* = 4 for each condition). Proteins were not appreciably depleted in any of the conditions. Error bars indicate SD. **(E and F)** FLS length distribution (E) and FLS count (F) from assays incubated for 20 min as usual (“Before”, *n* = 3,010) and by reusing the reaction mix from an assay to start another assay (“After”, *n* = 2,905). FLS length shows an approximate exponential distribution in both cases, and the numbers of FLSs were not reduced. Horizontal lines in F indicate the mean count. **(G–J)** Example images from FLS assays with actin (magenta) and immunostained N-WASP (G), VASP (H), Ena (I), and Fascin (J; all cyan). Scale tripod indicates 10 µm along each axis.

We extracted the fluorescence intensities of actin regulatory proteins at the tip complex and corresponding FLS morphologies using a custom image analysis pipeline FLSAce. FLSs appeared throughout the experiment, with the highest rate of appearance occurring a few minutes after the addition of extracts to the membrane (Fig. S2 A). The widest FLSs tended to nucleate early (Fig. S2 B), while the length was independent of nucleation time (Fig. S2 C). Protein incorporation into FLSs did not appreciably diminish the pool within the extracts, and FLSs could be renucleated by transfer of the extracts onto a fresh supported lipid bilayer (Fig. S2, D–F), suggesting that occlusion from other proteins on the supported bilayer limits their width at later time points, rather than depletion of components.

**Figure 3.**
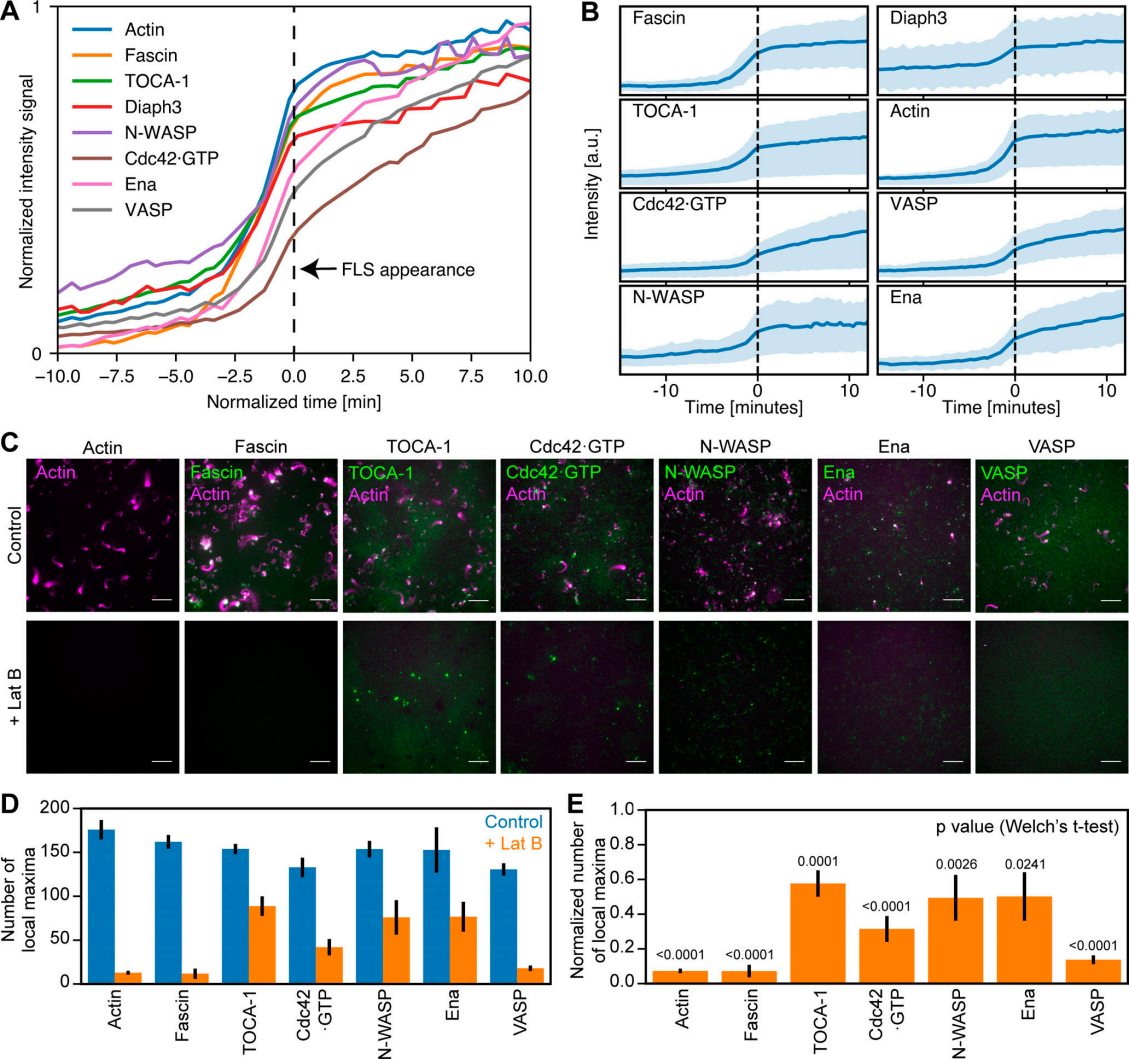
**Actin regulatory proteins assemble cooperatively to polymerize actin bundles.** **(A)** Mean intensity of accumulation in actin regulators over time as FLSs form combined from the experiments listed in Table 1: Actin *n* = 19,974; Fascin *n* = 2,898; TOCA-1 *n* = 19,893; Diaph3 *n* = 3,811; N-WASP = 1,743; Cdc42•GTP *n* = 7,839; Ena *n* = 1,828; and VASP *n* = 1,857. **(B)** Shaded area is the standard deviation of protein accumulation data from A. **(C)** LatB treatment reduces TOCA-1, Ena, and N-WASP foci on the membrane and inhibits recruitment of fascin and VASP, with partial inhibition of Cdc42•GTP localization. Scale bars = 10 µm. **(D)** Quantification of number of local maxima computed by protein fluorescence intensities plus and minus LatB. Error bars are the SEM. FLSs are from 8–15 fields of view of more than three independent experiments, and a normal distribution was assumed. **(E)**. The fraction of local maxima when LatB was added normalized to the control. P values are from two-sided Welch’s *t* test. Error bars represent SEM.

We tracked the accumulation of regulatory proteins relative to the time when FLSs reach 1 µm length, which we set as time zero. Assembly of the actin regulators to the tip complex occurs cooperatively, as indicated by the sigmoidal shape of their intensity curves (Fig. 3 A). Recruitment of the Cdc42•GTP probe GBD was delayed relative to the other proteins, likely due to competition with endogenous N-WASP. As we had observed in vivo, there was a striking heterogeneity in the mean fluorescence intensity of each component at steady state (Fig. 3 B). We confirmed that this was not due to compensation by untagged proteins by immunostaining endogenous Ena, VASP, and Fascin present in the extracts, where we saw similar heterogeneity (Fig. S2, G–J).

Due to the cooperative accumulation of proteins at the same time as the highest rate of increase in actin intensity (Fig. 3 A), we tested which of our proteins still assembled in the presence of the actin monomer sequestering drug latrunculin B (LatB). While TOCA-1 is still bound to the membrane in the presence of LatB, consistent with previous observations (Lee et al., 2010), the number of TOCA-1 foci was reduced by ∼40% in the absence of F-actin, with comparable reductions of Ena and N-WASP (Fig. 3, C–E). The recruitment of Fascin and VASP was prevented, similar to previous observations with Diaph3 (mDia2; Lee et al., 2010). The number of Cdc42•GTP foci at the membrane was also reduced by ∼70% (Fig. 3, C–E). These data are consistent with a role for F-actin patches in the assembly of FLS tip complexes and the convergent elongation model of filopodium formation (Svitkina et al., 2003).

**Figure 4.**
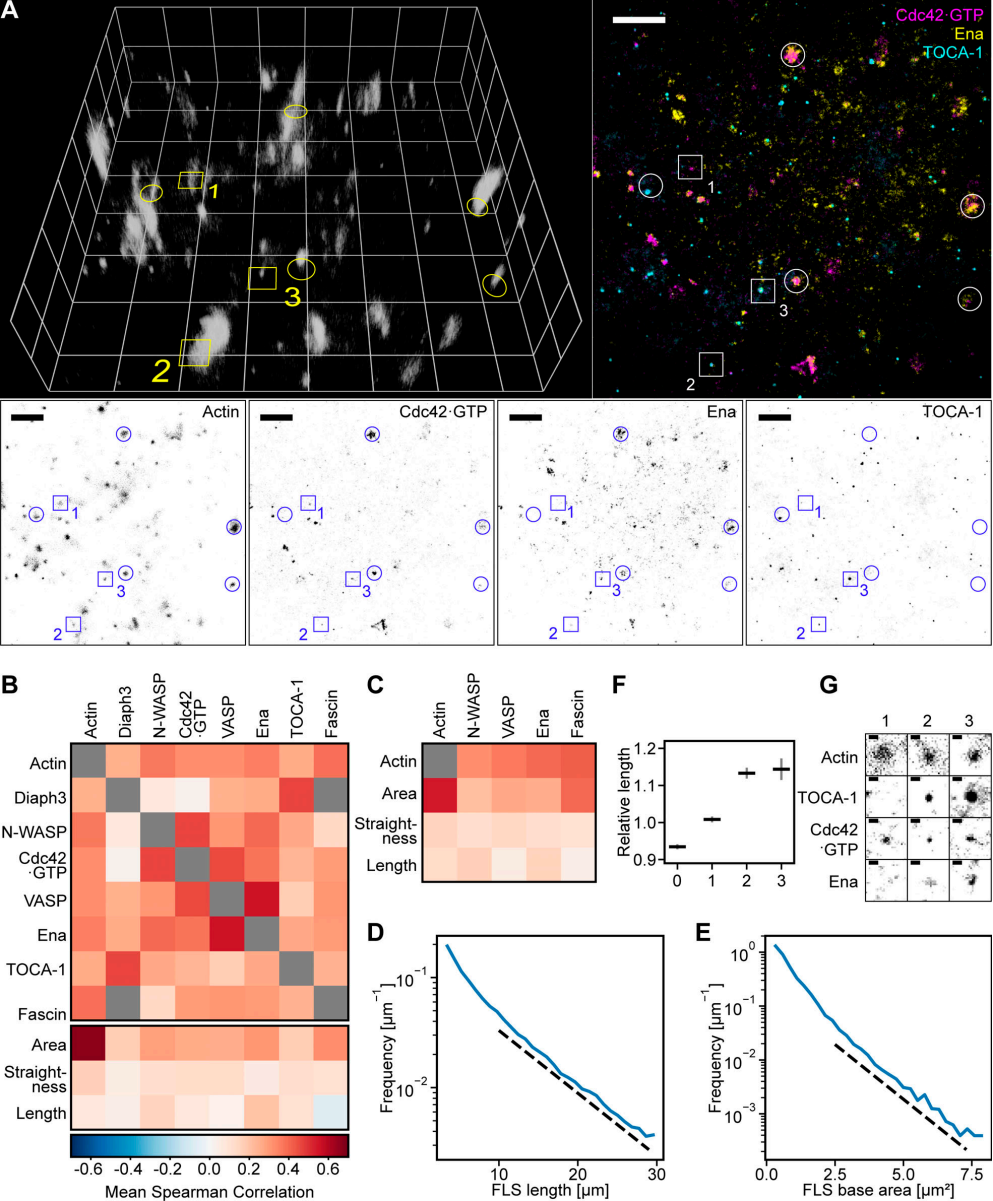
**Actin regulators are heterogeneous, display redundancy, and exhibit preferred subcomplexes.** **(A)** Complexes of diverse composition generate FLSs in vitro. Example of three different actin regulatory proteins (TOCA-1, Cdc42•GTP, and Ena) at the membrane together with a 3D reconstruction of the z-stack of the FLSs growing from a supported bilayer. Note the diversity of regulatory protein combinations observed under the FLSs (colored image in the top right, FLSs highlighted with circles throughout the panel). Grid spacing and scale bars = 10 µm. Volume-rendered FLSs are shown by setting each voxel’s transparency according to its measured intensity (lighter indicates higher intensity). Numbered squares indicate the positions of the example areas displayed in G. **(B)** Matrix showing Spearman protein–protein intensity correlation and morphology–protein intensity correlation values calculated separately per field of view and averaged. See Fig. S3 for FLS *n* numbers and Table 1 and Table 2 for numbers of experiments. Gray boxes mean exact correspondence (Diaph3 and Fascin are both enhanced GFP tagged). **(C)** Correlations of protein immunostaining intensity with actin intensity, FLS tip complex area, FLS straightness, and length are similar to the correlations from tagged protein experiments in Fig. 2 B. FLS numbers: Ena, *n* = 1,176; N-WASP, *n* = 1,971; VASP, *n* = 698; and Fascin, *n* = 877. **(D)** FLS lengths are approximately exponentially distributed. The dashed line is a guide for the eye and indicates an exponential with characteristic length L* = 7.6 µm. FLS count, *n* = 117,365. **(E)** FLS base areas are approximately exponentially distributed. The dashed line indicates an exponential with characteristic area A* = 1.07 µm^2^. FLS count, *n* = 117,365. **(F)** Relative change in FLS length for enriched FLS with zero, one, two, or three observed proteins compared with the total population length mean, calculated for each field of view and subsequently averaged. Error bars indicate the 95% confidence interval of the mean. Data were pooled from all the different combinations of proteins. Zero, *n* = 579; one, *n* = 577; two, *n* = 566; three, *n* = 376. **(G)** Closeups of individual tip complexes (from the numbered examples in A) showing cases where one, two, or all three proteins are enriched in an experiment containing labeled TOCA-1, Cdc42, and Ena. Scale bars = 1 µm.

We asked whether the heterogeneity we observed in protein intensities in different tip complexes was reflecting different subtypes of tip complex (e.g., Diaph3-driven, Fascin-driven, or Ena-driven FLSs). To do this, we performed a large series of experiments in which we examined triple combinations of our proteins of interest with the actin bundles visualized in 3D by wide-field microscopy in a fourth channel at steady state (Table 2). It was evident that actin bundles emerge from tip complexes with widely differing compositions (Fig. 4 A shows an example field of view from one combination). One possible explanation for such heterogeneity is an amplification of initial small protein number fluctuations by cooperative networks of protein interactions.

**Table 2.** Protein and fluorophore combinations used to measure steady-state tip complex compositions with three regulators

Protein 1	Fluorophore 1	Protein 2	Fluorophore 2	Protein 3	Fluorophore 3	Number of experiments	Fields of view
Diaph3	eGFP	GBD	mKate	SNAP–N-WASP	Alexa 647	4	22
Diaph3	eGFP	KCK-VASP	Alexa 568	SNAP-Ena	Alexa 647	4	27
Diaph3	eGFP	KCK-VASP	Alexa 568	SNAP–TOCA-1	Alexa 647	3	16
Fascin	eGFP	GBD	mKate	SNAP-Ena	Alexa 647	1	9
Fascin	eGFP	GBD	mKate	SNAP–TOCA-1	Alexa 647	4	41
Fascin	eGFP	GBD	mKate	SNAP-VASP	Alexa 647	4	40
Fascin	eGFP	KCK-VASP	Alexa 568	SNAP-Ena	Alexa 647	4	36
Fascin	eGFP	KCK-VASP	Alexa 568	SNAP–N-WASP	Alexa 647	4	36
SNAP–N-WASP	Alexa 488	GBD	mKate	SNAP–TOCA-1	Alexa 647	3	33
SNAP–N-WASP	Alexa 488	KCK-VASP	Alexa 568	SNAP-ENA	Alexa 647	4	42
SNAP–TOCA-1	Alexa 488	GBD	mKate	SNAP-ENA	Alexa 647	4	45
SNAP–TOCA-1	Alexa 488	GBD	mKate	SNAP–N-WASP	Alexa 647	4	39

eGFP, enhanced GFP.

**Figure S3.**
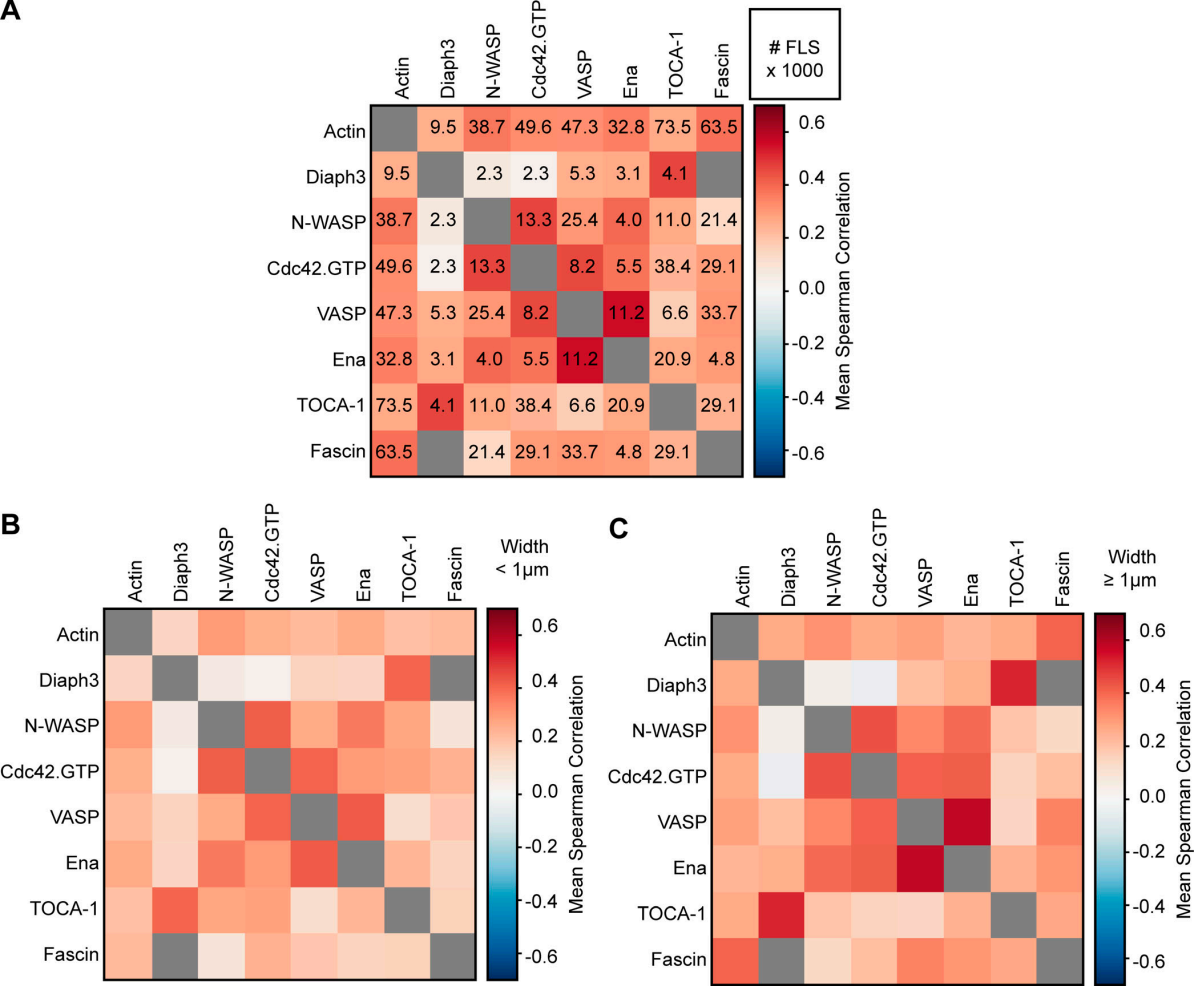
**Positive correlations between actin regulators.** **(A)** The correlation value matrix from Fig. 2 B with numbers of FLSs (lower right, in thousands). **(B)** Correlations between pairs of actin regulatory proteins for FLS tip complexes with an effective diameter below 1 μm. The color scale is the same as in Fig. 4 B. **(C)** Same as above for tip complexes with a diameter above 1 μm. The pattern of weak and stronger correlations is the same for both larger and smaller FLSs.

We pooled our two datasets to complete a matrix of correlations of each protein with every other protein and with morphology (Fig. 4 B, Fig. S3 A, Table 1, and Table 2). We found several protein pairs that exhibit strong intratip complex correlations: Ena/VASP, TOCA-1/Diaph3, VASP/Cdc42•GTP, and N-WASP/Cdc42•GTP. The high correlation between Ena and VASP can be explained by their known ability to form heterotetramers (Riquelme et al., 2015), and VASP was also reported to cooperate with Cdc42, though this was via IRSp53 (Disanza et al., 2013). Diaph3 is a recognized binding partner of TOCA-1 (Aspenström et al., 2006), and Cdc42•GTP activates N-WASP (Rohatgi et al., 1999). Nearly all other pairs showed weak positive correlations, with the exceptions of N-WASP/Diaph3 and Cdc42•GTP/Diaph3, which distribute randomly relative to each other (Fig. 4 B). Partitioning FLSs by size (less or more than 1-µm diameter) revealed the same patterns of correlations (Fig. S3, B and C). The overall positive correlation between most proteins suggests a cooperativity with more proteins joining the assembly when protein abundance increases, which agrees with the sigmoidal shape of protein assembly in the kinetic analysis (Fig. 2, C and D). These data ruled out the presence of discrete subtypes of FLS tip complex driven by distinct proteins, as in that scenario there would have been both strong positive and strong negative correlation values. Thus, we find a generally permissive but not totally promiscuous association between actin regulators in vitro.

**Figure S4.**
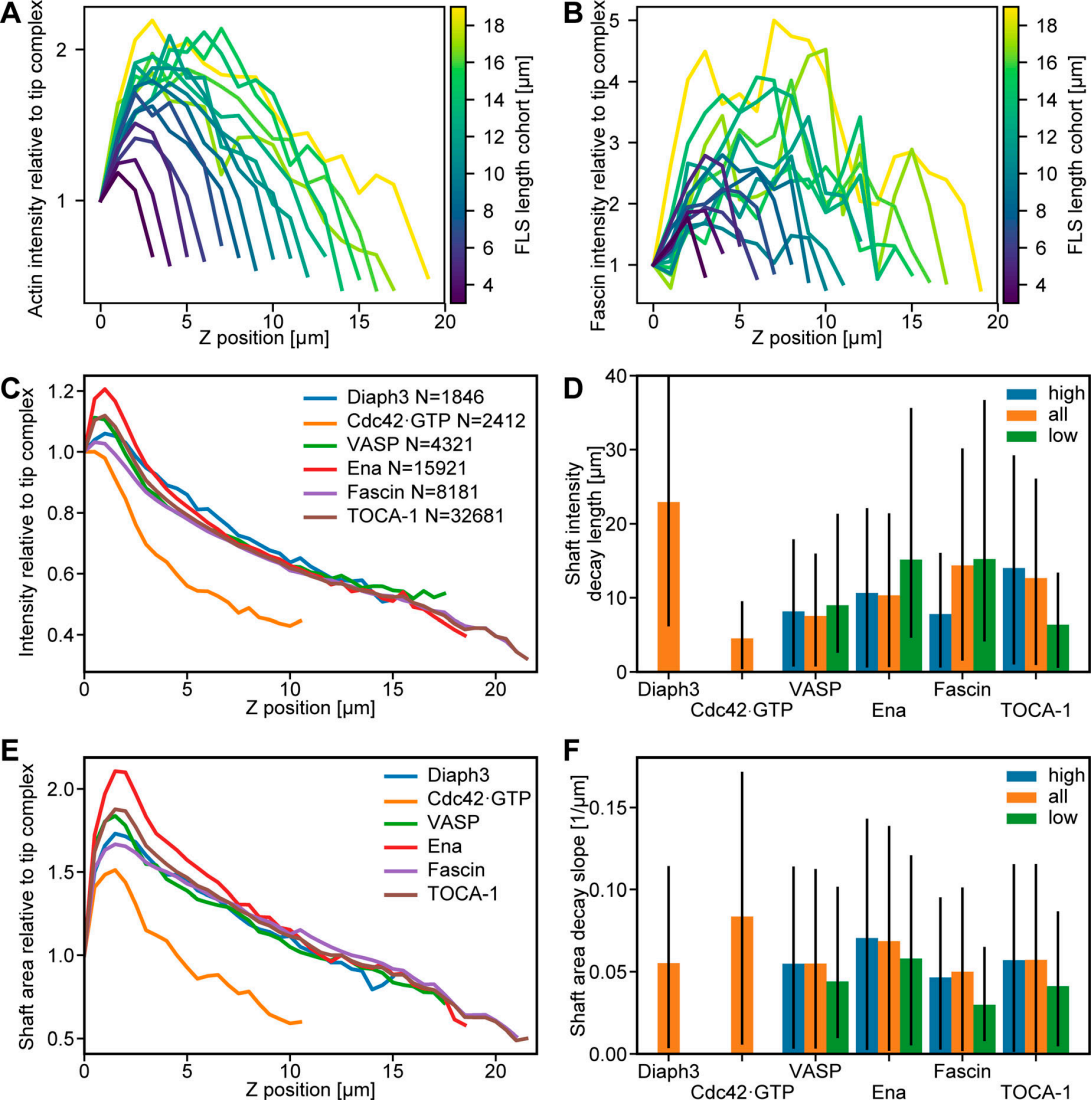
**The FLS shaft intensity is similar in all compositions.** **(A)** Actin intensity along the shaft perpendicular to the x-y plane and relative to the intensity at the FLS tip complex for FLS cohorts (*n* = 1,818). The FLSs are categorized according to their lengths in 1-μm cohorts. **(B)** The same as in A for the fascin intensity along the shaft. **(C)** Median actin intensity along the shaft perpendicular to the x-y plane and relative to the intensity at the FLS tip complex from assays with the given proteins labeled (*n* numbers given in the legend). **(D)** Median actin intensity exponential decay length along the FLS shaft from fitting an exponential decay for a given protein enriched (high) or absent (low) compared with the whole set (all). Diaph3/all, *n* = 1,846; Cdc42·GTP/all, *n* = 2,312; VASP/all, *n* = 4,321; VASP/high, *n* = 1,297; VASP/low, *n* = 1,297; Ena/all, *n* = 15,921; Ena/high, *n* = 4,797; Ena/low, *n* = 4,793; Fascin/all, *n* = 8,181; Fascin/high, *n* = 2,463; Fascin/low, *n* = 2,461; TOCA-1/all, *n* = 32,581; TOCA-1/high, *n* = 9,813; and TOCA-1 low, *n* = 9,806. Error bars represent the 66% confidence interval obtained via resampled residuals bootstrap. Missing bars are due to fitting failures resulting from too small *n* numbers. **(E)** Median cross-sectional FLS area along the shaft from assays with different proteins labeled (*n* numbers given in C). **(F)** Median negative slope of actin width from linear decay fits for a given protein enriched (high, above 70th percentile) or absent (low, below 30th percentile) compared with the whole set (all). *N* numbers given for D. Error bars represent the 66% confidence interval obtained via resampled residuals bootstrap.

**Figure S5.**
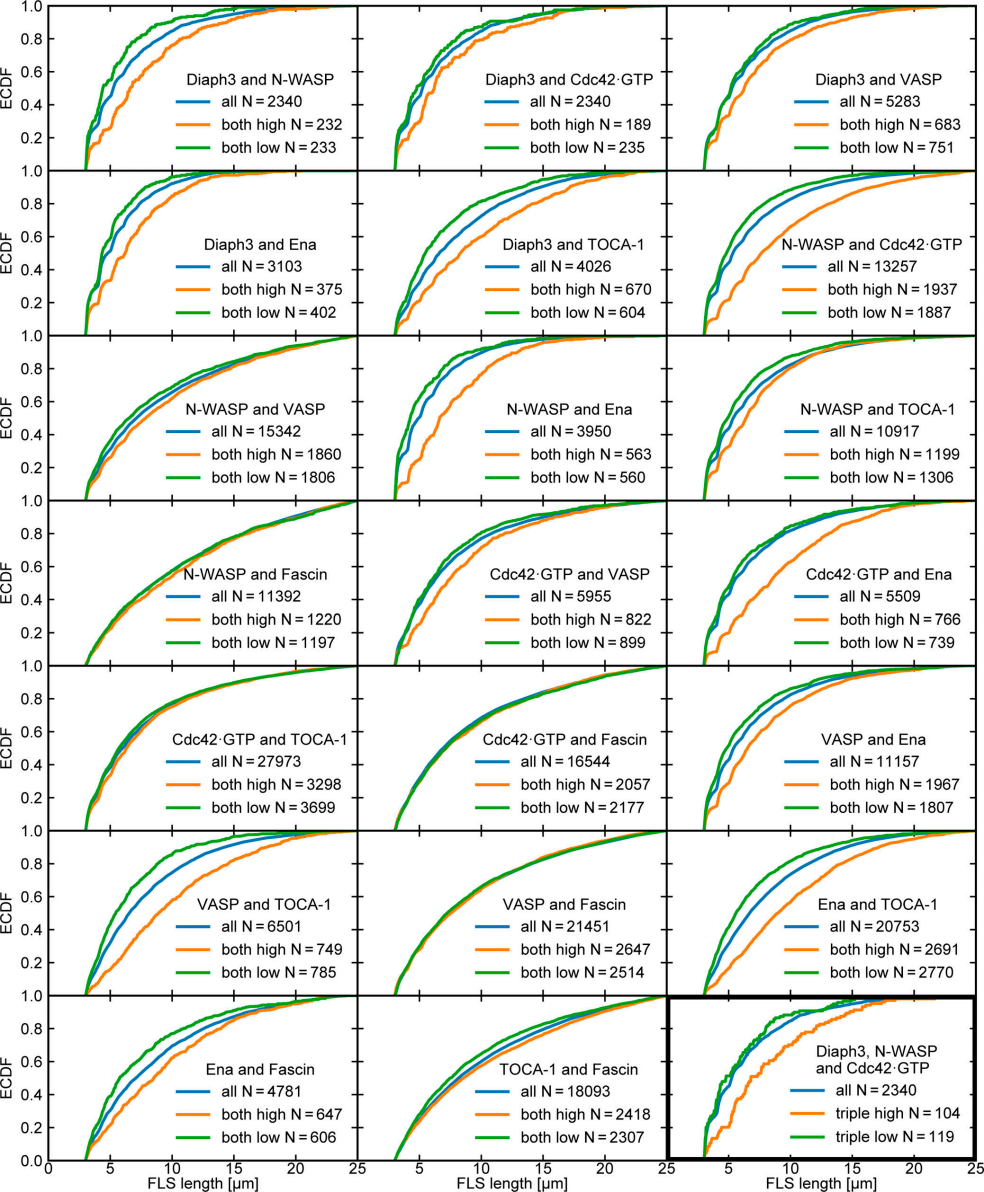
**FLS lengths for different double combinations of proteins.** Cumulative frequency plots (empirical cumulative distribution function, ECDF) of FLS length for all double combinations of regulatory proteins for the specific cohort (blue lines), both proteins enriched above a 70% intensity threshold (orange lines), and both proteins below the enrichment threshold (green lines). *N* values are given in the panel legends. All combinations enriched in Diaph3 and Ena are longer, together with some others. FLSs enriched in Fascin are usually no longer than those without, except when TOCA-1 is also enriched (compare green and orange lines). The triple combination of Diaph3/N-WASP/Cdc42•GTP shows similar effects (bottom right, black outline). Overall, length arises from multiple small, interacting effects.

We found a very weak positive correlation between each of the protein intensities and the area of the tip complex, FLS straightness, and actin intensity, as well as FLS length, which we observed with both protein addition and immunostaining (Fig. 4 B; Fig. 4 C; and Fig. S2, G–J). The correlations were similar in magnitude to our observations in *Drosophila* (Fig. 1 J–L). Also similarly to filopodia, FLS lengths are exponentially distributed (Fig. 4 D), as well as the tip complex areas (Fig. 4 E). We saw no difference in the shaft intensity of actin except when GBD was used as a probe, where actin intensity is reduced presumably because it reduces the available levels of Cdc42•GTP (Fig. S4). FLSs emerging from tip complexes simultaneously enriched (in the top half of above-background fluorescence intensity values) with at least two of the observed regulators were 10% longer than those with none or one regulator. (Fig. 4, F and G shows example FLSs with one, two, or three regulators enriched, taken from Fig. 4 A.) Examination of the specific combinations of any two enriched proteins revealed that FLSs with high levels of Diaph3 or Ena plus any other protein were longer than FLSs overall (compare orange and blue lines in Fig. S5). Some other specific pairs of enriched proteins also showed increased length (e.g., N-WASP and Cdc42•GTP), showing that Diaph3 and Ena are not exclusive in their influence. The specific triple combination enriched in Diaph3 + Cdc42•GTP + N-WASP was similar to data from the pairs (Fig. S5). Therefore, the small increase in length arises from multiple pairs of protein combinations.

### FLS growth dynamics and theoretical framework

**Figure 5.**
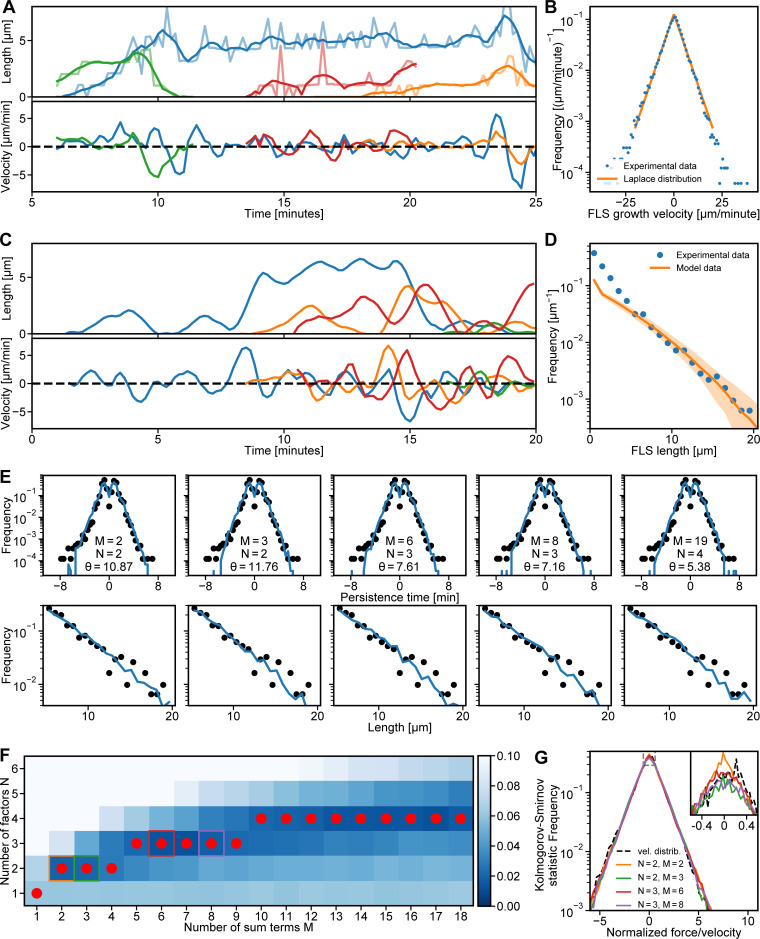
**Mathematical framework for FLS growth captures experimental FLS length distribution and growth dynamics.** **(A)** Four smoothed, different example experimental FLS length trajectories (top) and their instantaneous growth velocities (bottom) in different colors. **(B)** Histogram of measured FLS growth velocities (*n* = 114,816) from tracked FLS trajectories with maximum likelihood estimation fit of a bi-exponential Laplace distribution. **(C)** Four randomly selected smoothed example trajectories (top) generated from our sum-product framework with their instantaneous velocities (bottom). We started simulations at random initiation points at 0–20 min. **(D)** FLS length distribution (blue circles; *n* = 3,193 observations) and corresponding data from simulating 10,000 trajectories (solid orange line; shaded area is the 95% confidence interval of the histogram bin means). The simulation data histogram was scaled by the ratio between the median FLS lengths larger than 5 µm and the median simulated FLS lengths larger than 5 µm to visualize agreement between theory and experiment. **(E)** Predicted persistence time distribution (top row, blue solid lines) and length distributions (bottom row, blue solid lines) agree with the experimental distributions (black dots) for the different values of *M* and *N* in Fig. 3 E. Values for θ are given in min^−1^. Persistence time distribution *n* = 20,679. **(F)** Comparison of the experimental normalized growth velocity distributions to the simulations for different combinations of number of sum terms *M* and number of product factors *N*, using the Kolmogorov-Smirnov goodness-of-fit statistic. Darker blue indicates a better fit. The best fit *N* for any given *M* is highlighted by a red dot and follows an approximate square root dependence. Colored squares correspond to the data shown in G. **(G)** Force/velocity distributions (vel. distrib.) for the five combinations highlighted by colored squares in F. The dashed line is the normalized experimental growth velocity. The inset shows an enlargement of the peak of the distributions indicated by the dashed rectangle. The data used to generate the graphs is available in the Supplemental data.

We next examined the dynamical properties of FLS length using high time resolution confocal microscopy. Careful filtering for segmentation errors and discontinuities allowed us to extract the growth velocities (Fig. 5 A). The resulting time series shows an initial rapid growth phase followed by erratic cycles between elongation and shrinkage (Fig. 5 A and Video 7). Our analysis resembled previous reports of lamellipodial dynamics (Betz et al., 2006). Given the large numbers of proteins involved in the filopodium regulatory machinery and the resulting complex interaction networks, we had expected to see highly complex growth dynamics. However, we found that the histogram of growth velocities (of all FLS time series) had largely symmetric exponential tails in both positive (growth) and negative (shrinkage) directions and a mean of approximately zero (Fig. 5 B). The Laplace distribution fit the data extremely well (Fig. 5 B), and like the exponential distribution, it was described by a single parameter, its variance. This indicated that, at some point in the actin polymerization process, the molecular complexity in the regulatory machinery is reduced or obscured such that there is a single overall controlling variable. An analogy would be how microscopic molecular interactions in gases give rise to temperature as a macroscopic observable property.

**Video 7.** **Time-lapse z-stack spinning disk confocal video of actin visualized with GFP-utrophin CH domain probe at 20–30 min. **The video shows individual FLSs oscillating between growth and shrinkage phase. Scale bar represents 10 µm. Images were captured at 10-s intervals and are replayed at 6 frames per second.

We sought to understand how this growth velocity distribution could apparently arise from the complex networks of molecular associations observed between the actin regulatory proteins at the tip complex. Our long-tailed exponential length distributions suggest that stochastic processes are governing filopodial dynamics. However, simple monomer addition and removal processes do not yield a Laplace distribution of growth velocities; they yield normal distributed growth velocities.

Mathematically, a Laplace distribution emerges when four normally distributed random variables $X_{1},X_{2},X_{3}$, and $\;X_{4}\;$are combined as the sum of products $V=X_{1}X_{2}+X_{3}X_{4}$ (see Mathematical analysis section). Here, we are interpreting the variable *V* as the instantaneous growth velocity of a given FLS. The *X*s are the (normally distributed) fluctuations around baseline concentrations of actin regulatory proteins at the tip complex. Product terms arise from complex-forming molecular reactions $X_{1}+X_{2}\to X_{1}X_{2}$, with the complex $X_{1}X_{2}$ producing FLS elongation or shrinkage, while the sums represent the independent contributions of two complexes $X_{1}X_{2}$ and $X_{3}X_{4}$ to growth velocity. The dynamics of the concentration fluctuations *X* are controlled by a single parameter, the relaxation rate θ, which determines how quickly a given protein concentration returns to its baseline after a random change. Together, this provides a framework that explains the appearance of the Laplace distribution in the growth dynamics of FLSs from stochastic regulator concentration fluctuations. We used the framework to simulate growth velocity time series and, via numerical integration, FLS length time series, resembling those obtained experimentally (Fig. 5 C). We collected length data points at the 20-min time point, both in our simulations and in experimental data. The resulting length distributions were well matched (Fig. 5 D), with only the single parameter θ needing to be fit. The framework also reproduced the shape of the distribution of persistence time for the FLS growth/shrinkage phases (Fig. 5 E and Video 7). The one exception was a mismatch in the distribution for small FLS lengths, which was due to the initial start of FLS growth not being captured by our theory.

Our theoretical framework indicated that after initiation, which we know is Arp2/3 complex dependent (Lee et al., 2010), FLS growth is governed by multiple redundant mechanisms. Strictly interpreted, the minimal requirement is that two independent complexes consisting of two regulatory protein pairs control the growth and shrinkage dynamics of actin structures. The correlation data in Fig. 4 B, however, indicates that there are at least three pairs of two regulators each, namely N-WASP/Cdc42•GTP, TOCA-1/Diaph3, and Ena/VASP. We found that combinations of *M* complexes composed of *N* proteins also give rise to a Laplace-shaped velocity distribution when $N\approx \sqrt{M}$ (Fig. 5, F and G). Hence, the growth dynamics we observed can generically arise from a multitude of amalgamations of actin regulators. The minimal theory compatible with our data (Fig. 4 B) consists of a sum of three pairs (the case of *M* = 2 and *N* = 3 in Fig. 5 F, green box). Indeed, this combination (and higher *N *and *M*) provides an arguably better fit to the data than the pure Laplace case of *M* = 2 and *N* = 2 (Fig. 5 F, orange box) since it accounts for the slight rounding of the peak around zero growth velocity (Fig. 5 G, inset).

### Testing the dynamics of regulators predicted by the theory

**Figure 6.**
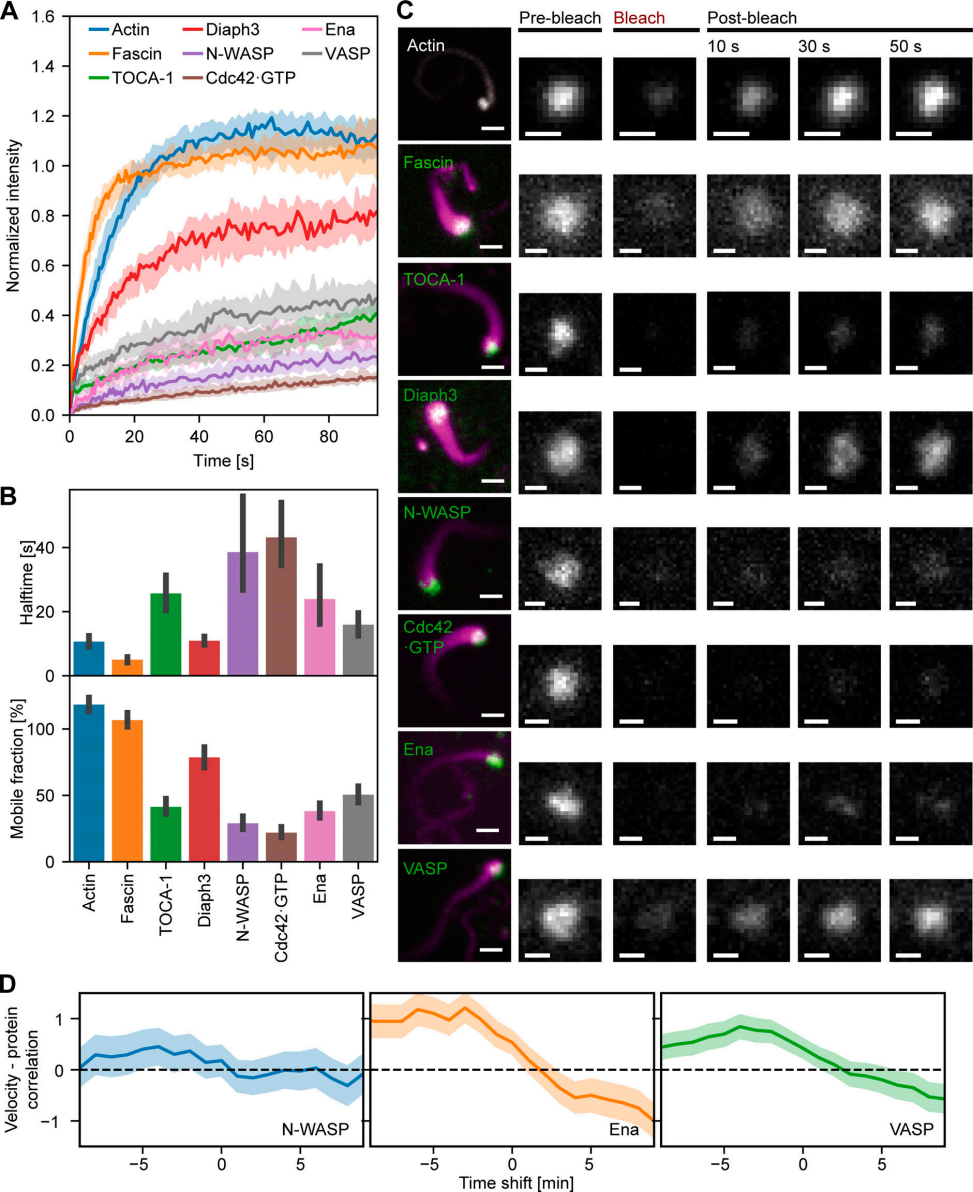
**The FLS tip complex actin regulatory protein is semidynamic.** **(A)** Time course recovery of AF488 actin (*n* = 65), GFP-Fascin (*n* = 40), AF488 TOCA-1(*n* = 51), GFP-Diaph3 (*n* = 34), AF488 N-WASP (*n* = 39), pmKate-GBD (*n* = 39), AF488 Ena (*n* = 36), and AF568 VASP (*n* = 51) after photobleaching in tips of FLSs at steady state grown for at least 30 min. Solid lines show the median, and the shaded area is the 95% confidence interval. **(B)** The half-time and percentage recovery from fitted exponential curves for each protein. Error bars represent the 95% confidence interval of the mean. **(C)** Actin regulatory protein localization to FLS and fluorescence recovery after photobleaching at FLS tips (green: protein of interest, magenta: AF647 actin). Protein concentrations unlabeled/labeled: Actin 210/4,000; TOCA-1 10/4; VASP 45/24; N-WASP 18/18; GBD 2.46/0; fascin 650/625; Diaph3 16/15; and Ena 60/60 in nanomolars. Scale bars = 2 µm (main images) or 1 µm (insets). **(D)** Time series cross-correlation of the absolute values of the FLS growth velocity and background-corrected N-WASP (*n* = 780), Ena (*n* = 2,003), and VASP (*n* = 2,783) intensities at FLS tip complexes, averaged over all measured FLS trajectories. The graph shows the cross-correlation coefficient at the given time shift (negative time shifts mean that intensity changes precede velocity changes). Shaded areas are 95% confidence interval of the mean.

The theoretical framework relies on fluctuations within the concentrations of the components rather than their average concentrations. We therefore asked whether the FLS tip complex is dynamic by performing FRAP experiments (Fig. 6, A–C). A high immobile fraction is consistent with the nonzero baseline concentration of actin regulators in the tip complex required by the theory. The recovery half-times of the fastest components, Fascin, Diaph3, Ena, and VASP, were 5–25 s, agreeing with theoretical predictions (Fig. 6 B) and recent data for VASP in neurons (Boyer et al., 2020). For the minimal case of M=3 complexes of N=2 regulatory factors, we calculated how quickly the proteins would be fluctuating within the tip complex. Mathematically, the time scale of fluctuation τ scales as λ/N, where λ is the growth velocity relaxation rate. We estimate that $\lambda \approx 22\;min^{-1}$ (determined from the fitted values of relaxation rate θ in Fig. 5, assuming that θ is the same for all the proteins involved) gives a relaxation rate of around 11 min^−1^, which corresponds to a recovery half-time of ∼5 s. This is the same order of magnitude as in our observations and matches the fastest components, which would dominate in practice. In addition, the high-correlation pairings N-WASP/Cdc42•GTP and Ena/VASP (Fig. 4 B) showed similar percentage recovery after photobleaching, consistent with their physical interaction (Fig. 6, A and B; and Fig. 4 B). Diaph3 had higher mobility with lower half-time than its highest correlating partner, TOCA-1, suggesting that other interactions of TOCA-1 and Diaph3 control their residence time in the complex, such as TOCA-1 with the membrane and Diaph3 with F-actin (Lee et al., 2010).

The theory predicts that a change in filopodial growth velocity has to be preceded by a fluctuation of a regulator. To test this, we measured rapid time-lapse stacks of the actin bundle growth and shrinkage at steady state with the fluorescence intensity of Ena, VASP, and N-WASP. We tracked the correlations between the absolute growth velocity and the time-shifted absolute protein intensity level. As expected, there was now a positive correlation between actin growth activity and protein intensity, with the maximal correlation at a time offset of ∼4 min (Fig. 6 D; shaded areas are the 95% confidence intervals). This offset is consistent with the need for actin incorporation to occur between the protein appearing and a resulting change in FLS length. The strength of correlation for N-WASP is lower than for Ena or VASP, presumably because it is more upstream as an Arp2/3 complex nucleation promoting factor rather than a barbed-end polymerase such as Ena and VASP. The negative correlations after *t* = 0 showed that a high change in velocity also precedes a change in protein levels at the tip complex, suggesting some sort of mechanical feedback on the molecular assembly of the tip complex. This is not a feature covered by the theory but agrees with actin regulators moving in and out of the tip complex in response to actin polymerization.

### Fascin localization is heterogeneous, and its excess causes a deviation from exponential length distributions in *Drosophila*

**Figure 7.**
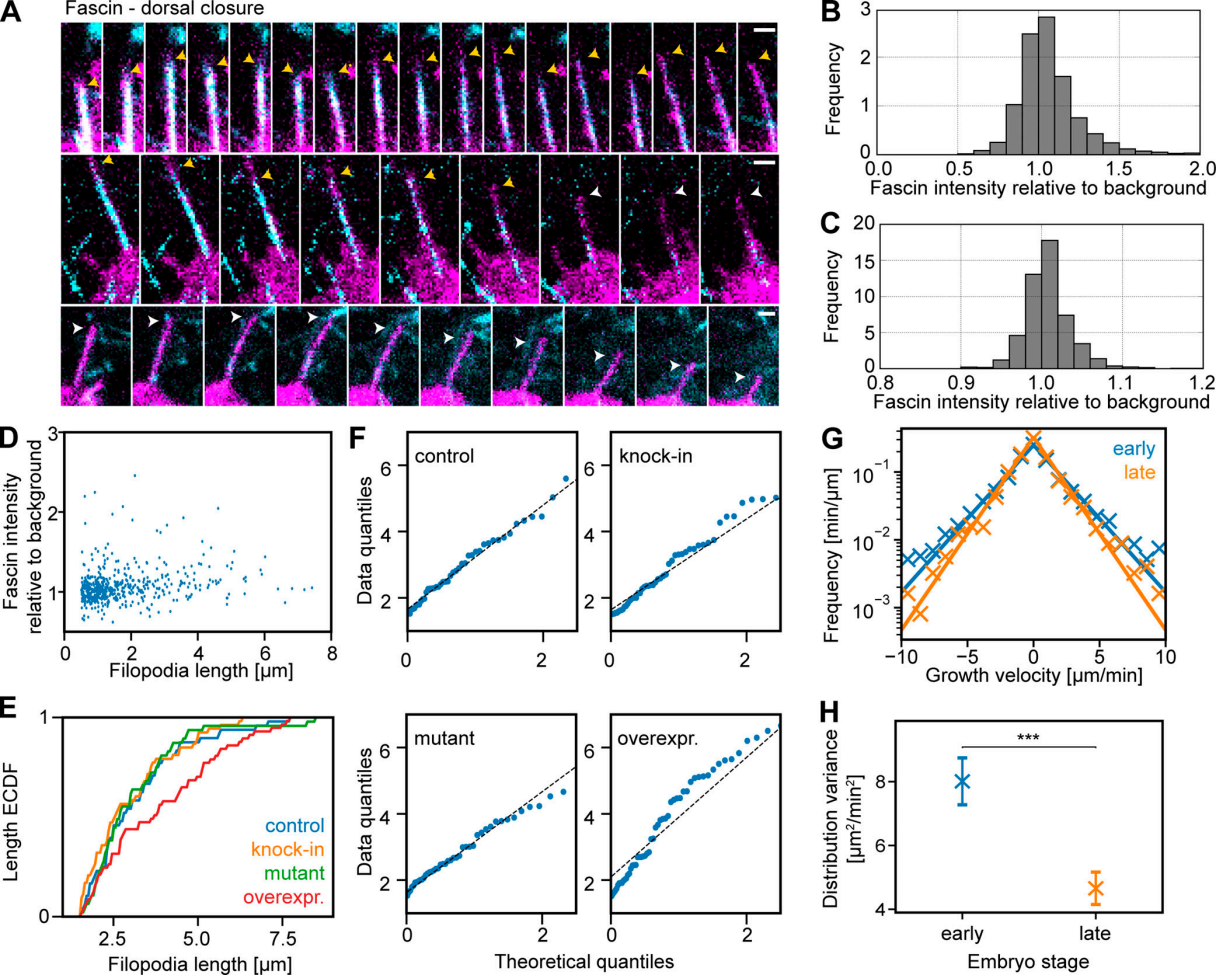
**Heterogeneous levels of fascin and corresponding length distributions in vivo.** **(A)** Time-lapse montages of maximum-intensity projections every 15 s of filopodia in leading edge cells in dorsal closure in the *Drosophila* embryo. Endogenous fluorescent Fascin is in cyan and membrane marker in magenta. Yellow and white arrowheads indicate filopodia with and without fascin. Scale bars = 1 µm. **(B)** Histogram of Fascin fluorescence intensity normalized to local background in filopodia, set to 1 (*n* = 3,432). **(C)** Histogram of Fascin fluorescence intensity normalized to local background in FLS shafts (*n* = 1,515). Most filopodia and FLSs only show background levels of fluorescence, with few at higher intensities. **(D)** Scatterplot of fascin intensity in filopodia shafts versus maximal filopodia length in leading edge cells in dorsal closure (*n* = 519). **(E)** Cumulative frequency plot (empirical cumulative distribution function, ECDF) of filopodia lengths with mutant (*n* = 47), wild-type (*n* = 48), knock-in GFP-Fascin (*n* = 53), and overexpressed (overexpr.) GFP-Fascin (*n* = 57) in dorsal closure leading edge filopodia. P values compared with control with two-sample Kolmogorov-Smirnov test: labeled Fascin, P = 0.765; mutant Fascin, P = 0.996; and Fascin overexpression, P = 0.047. **(F)** Q:Q plots show that control, labeled (knock-in) fascin, and mutant fascin filopodial lengths are consistent with an exponential distribution, but Fascin overexpression is not. **(G)** Lateral transverse myotube filopodial growth velocity distribution in stage 15 (early, *n* = 191) and stage 16 (late, *n* = 121). Crosses indicate experimentally derived data, and solid lines show maximum likelihood fits of a Laplace distribution. **(H)** Laplace distribution variances shown as crosses. Error bars indicate the 95% confidence intervals. The distributions for early and late stages are significantly different (***P = 0.00028, two-sample Kolmogorov-Smirnov test). Data are from four time-lapse movies each. Colors are the same as in G.

In FLSs, we measured Fascin at the tips; however, Fascin is also present in the shaft of both FLSs and filopodia. Since the theoretical framework does not rely on a tip localization, we used the behavior of Fascin in the shaft as a test of our in vitro findings. Consistent with all of our other observations, filopodia had vastly differing intensities of Fascin in their shafts (Fig. 7 A). We measured maximal Fascin intensities through the shaft of FLSs and filopodia in dorsal closure, finding comparable distributions of intensities (Fig. 7, B and C). This indicated a similar function of fascin in both FLSs and filopodia, despite the different spatial constraints of the membrane. Fascin comes and goes in the shaft of filopodia, originating from the tip, in agreement with a fluctuation-based model (Video 8). Similar to our results with Ena and Scar at filopodia tips, we saw no correlation between the maximal lengths of filopodia and their intensity of Fascin (Fig. 7 D). In *Drosophila*, there was little effect on the mean lengths of filopodia from reducing the levels of fascin, where the loss of both fascin and the alternative bundling protein forked has a greater effect (Okenve-Ramos and Llimargas, 2014). Our framework predicts that the lengths of Fascin-null filopodia should be exponentially distributed but that overexpression should lead to a deviation in the length distribution from exponential, as this would flood a fluctuation-based mechanism, making the effect of a single complex dominant (mathematically equivalent to setting *M* = 1).

**Video 8.** **Maximum-intensity projections of laser scanning confocal time-lapse z-stack of *GFP-fascin, en-Gal4;UAS-cd8mCherry* embryo, stage 15.** Representative video of 17 from 14 embryos. Scale bars represent 10 µm. Images were captured at 15-s intervals and are replayed at 10 frames per second.

To quantify the effects of perturbing Fascin, we measured the length distributions of filopodia during *Drosophila* dorsal closure under conditions of loss of Fascin using the *singed* mutant or gain of Fascin by overexpression of GFP-Fascin using the engrailed-GAL4 driver by comparison with the GFP knock-in Fascin. The length distribution of dorsal closure filopodia from control embryos, GFP-Fascin knock-in filopodia, and filopodia in the Fascin mutant embryos was well fitted by exponentials (Fig. 7, E and F). In agreement with the theory, overexpression data deviated markedly from an exponential distribution, in contrast to all cases of filopodia length measurements we have examined so far (Fig. 7, E and F).

### Laplace-distributed filopodial growth dynamics in vivo

Our previous use of semiautomated methods to quantify filopodia in *Drosophila* myotube migration (Richier et al., 2018) gave us access to a rich dataset of manually annotated filopodia that could be used to train a machine learning algorithm to segment filopodia in tissue over time. In this tissue, we found that filopodia lengths were exponentially distributed. Moreover, there is a developmental transition where the myotubes attach to tendon cells within the wall of the *Drosophila* and the myotube filopodia are naturally suppressed (Richier et al., 2018). The filopodial growth and shrinkage velocities at early and late stages (where the data were independently acquired) were both well approximated by a Laplace distribution (Fig. 7 G), proving our results from the FLS system in vivo (Fig. 5 B). Comparing early and late stages, the width of the growth velocity distribution was reduced while retaining the shape of the Laplace distribution. This shows that the complex biochemical and cell biological machinery that operates within this developmental context acts to tune the single parameter that describes filopodial dynamics mathematically (Fig. 7 H). Together, these results demonstrate the biological relevance of the findings we identified in the cell-free system.

## Discussion

We have found that actin regulatory proteins form heterogeneous semidynamic assemblies on membranes composed of at least three or four different subcomplexes where actin bundles nucleate and grow. The resulting actin bundles grow and shrink with velocities that fall on a Laplace distribution, which results in exponentially distributed filopodial lengths. Using the mathematics governing probability distributions, we were able to link our observed velocity distributions to pairs of fluctuating actin regulators. The subcomplexes we identified are reminiscent of proteins and interactions that were previously thought to be important in filopodium formation. Cdc42•GTP was most highly correlated with VASP and N-WASP; (Disanza et al., 2013; Ma et al., 1998) Ena and VASP correlated with each other (Riquelme et al., 2015); and Diaph3, previously implicated in de novo filopodia nucleation, correlated with membrane-adaptor protein TOCA-1 (Aspenström et al., 2006), although not with Cdc42•GTP. However, with the complex composition of the extracts and multiple interaction partners for all the proteins involved, we do not yet conclude that no correlation means no relevant interaction.

Previous theoretical work considered actin filament length distributions resulting from monomer addition-removal processes together with fragmentation driven by gelsolin (Edelstein-Keshet and Ermentrout, 1998; Ermentrout and Edelstein-Keshet, 1998) and how length control can emerge from other properties of cytoskeletal regulation (such as limited monomer availability, active transport of monomers, capping protein, and formin inhibitors; Mohapatra et al., 2016; Mohapatra et al., 2015; Zhuravlev and Papoian, 2009). In contrast, we observed long-tailed exponential length distributions both in vitro and in vivo, suggesting that stochastic processes are governing filopodial dynamics. Our primary result shows that FLS and filopodial growth velocities follow a Laplace distribution. These observations are not compatible with simple monomer addition/removal processes, yet still point to a simple emergent dynamic arising from molecular complexity. The fluctuations of components on which the theory depends may originate from many different biochemical possibilities. For example, ubiquitination cycles of VASP have been observed to alter its dynamics within the tip complex, together with filopodial properties, downstream of netrin-1 signaling (Boyer et al., 2020). Other possible molecular candidates include phosphorylation cycles, GTP/GDP exchanges, or specific protein–protein interactions.

The heterogeneity we report resembles observations made for clathrin-mediated endocytosis in mammalian cells and components of the adhesome present in filopodia (Jacquemet et al., 2019; Taylor et al., 2011), suggesting that a similar mechanism based on a heterogeneity of multiple players is a more general feature of cell regulation. The redundancy in molecular composition allows a robustness and may also allow a variety of upstream and downstream components to intersect with the control of filopodia and co-opt them in diverse biological contexts. A multicomponent system could also ensure that signals regulating filopodia must be multiple and coincident, as only rarely will a single input be sufficient to cause an effect, and it takes an overexpression scenario to subvert the normal homeostatic mechanisms, such as Fascin in cancer (Tan et al., 2013). In FLSs, the membrane interactions together with SH3 domain and proline-rich regions in Ena, N-WASP, VASP, and Diaph3 are similar to observations with N-WASP and Nck in purified systems that have phase separation properties (Banjade and Rosen, 2014; Li et al., 2012). It may be that a Laplace-distributed output and the harnessing of fluctuations is the reason for such organization. We show here that in spite of a dynamic and heterogeneous tip complex, a constraint emerges in the resulting activity, which may be what allows actin machinery to be co-opted in a stereotypical manner, accommodating different tissue regulatory programs without any alteration to its underlying functional properties.

## Materials and methods

### Plasmids

*Xenopus tropicalis* TOCA-1 (GenBank accession no. BC080954) was PCR amplified from IMAGE clone 6980375 (Source Bioscience), with oligos of sequence 5′-GCA​TGG​CCG​GCC​ACC​ATG​AGC​TGG​GGT​ACT​G-3′ and 5′-GGC​GCG​CCT​TAG​ATA​TAA​GTT​ACT​GC-3′ using Phusion High-Fidelity DNA Polymerase and cloned into pET SNAP precision FA. *X. tropicalis* N-WASP (WASL; GenBank accession no. BC067309) was PCR amplified from IMAGE clone 5379332 (Source Bioscience), with oligos of sequence 5′-GAT​CGG​CCG​GCC​AAT​GAG​TAA​CAA​TA-3′ and 5′-GAT​CGG​CGC​GCC​TCA​ATC​CTC​CCA​TT-3′, and cloned into pCS2-his-SNAP-FA. ZZ-TEV-WIP (Ho et al., 2004) was a kind gift from the Kirschner Laboratory (Harvard University, Cambridge, MA). *Xenopus laevis* VASP (GenBank accession no. BC077932) was PCR amplified from IMAGE clone 5515353 with additional residues KCK added before the ATG, with oligos of sequence 5′-GCA​TGG​CCG​GCC​TAA​GTG​CAA​GAT​GAG​TGA​GAC​AGC​ACT​GG-3′ and 5′-GGC​GCG​CCG​GTC​AAG​GAG​TAC​CC-3′, and cloned into pET SNAP precision FA. Site-directed mutagenesis with oligos 5′-GGT​TGT​AAT​AAA​CTC​CCC​ACT​GGT​GAG​AG-3′ and 5′-CTC​TCA​CCA​GTG​GGG​AGT​TTA​TTA​CAA​CC-3′ was performed to destroy endogenous cysteine (conversion to serine at amino acid 63). *X. laevis* Ena (GenBank accession no. BC073107) was PCR amplified from IMAGE clone 5379332, with oligos of sequence 5′-GCA​TGG​CCG​GCC​ACC​ATG​AGT​GAA​CAG​AGC​ATC-3′ and 5′-GGC​GCG​CCC​TAT​GCG​CTG​TTT​G-3′, and cloned into pCS2 his SNAP acceptor. *X. laevis* Fascin (GenBank accession no. BC097600) was PCR amplified from IMAGE clone 4970584, with oligos 5′-GCA​TGG​CCG​GCC​ACC​ATG​AGT​TCT​GGA​CCC-3′ and 5′-GGC​GCG​CCT​TAG​TAT​TCC​CAG​AG-3′, and cloned into pCS2 his GFP FA (GFP was PCR amplified flanked with ecoRI/fseI restriction sites using oligos of sequence 5′-GCA​TGA​ATT​CAC​CAT​GGT​GAG​CAA​GGG​C-3′ and 5′-GAT​CGG​CCG​GCC​GGA​TCC​CCT​TG-3′ and cloned into pCS2 his FA acceptor). Human N-WASP GBD domain was PCR amplified from pCS2-mRFP-GBD (a kind gift from William Bement, Addgene plasmid 26733), with XhoI/AscI flanking restriction sites using oligos of sequence 5′-GAT​CCT​CGA​GCC​GGA​CCT​AGC​CCA​GC-3′ and 5′-GGC​GCG​CCT​CAC​TCC​TGG​CGC​CTC​ATC-3′, and cloned into pET-pmKate2. *X. laevis* Diaph3 was synthesized by Life Technologies based on sequence alignments made with Mayball cDNA information flanked with fseI/ascI restriction sites and cloned into pCS2 his GFP FA. *X. laevis* IRSp53 (GenBank accession no. BC108583) was PCR amplified from IMAGE clone 7981694 (Source Bioscience), with oligos of sequence 5′-GCA​TGG​CCG​GCC​ACC​ATG​TCC​CGG​GAC​GCA​G-3′ and 5′-GGC​GCG​CCT​CAT​CGG​ATG​ATT​GGC​G-3′ and cloned into pCS2-his-SNAP-FA.

### Protein purifications

Unless otherwise indicated, chemicals were purchased from Sigma-Aldrich, all steps were performed at 4°C, purified proteins were concentrated using a 10,000 MWCO spin concentrator (Millipore), their concentrations were determined based on their absorption at 280 nm, and they were stored at −80°C in 10% glycerol following snap freezing in liquid nitrogen. We expressed and purified *X. laevis* or *X.* *tropicalis* TOCA-1, the GBD of N-WASP, to monitor Cdc42•GTP (GBD), N-WASP, Ena, VASP, Diaph3, Fascin, and IRSp53. 6His-SNAP–TOCA-1, 6His-SNAP-Ena, 6His-SNAP–N-WASP/ZZ-WIP, and 6His-SNAP-IRSp53 were expressed as SNAP-fusion proteins. 6His-KCK-VASP was engineered to contain a cysteine close to the N-terminus for chemical labeling as previously published (Hansen and Mullins, 2010). 6His-GFP-Fascin and 6His-GFP-Diaph3 were engineered as N-terminal GFP-fusion proteins and 6His-pmKate-GBD as N-terminal fusion protein with red fluorescent protein pmKate2. We used commercially available actin labeled with Alexa647 (Life Technologies), Alexa568 (Life Technologies), and Atto390 (Hypermol). We also expressed the actin-binding 6His-GFP-utrophin-calponin homology domain as an actin probe for our high-speed microscopy time-lapse movies.

To purify 6His-SNAP–TOCA-1, 6His-mKate-GBD, 6His-SNAP-Ena, 6His-GFP-Fascin, 6His-GFP-utrophin-CH, 6His-GFP-Diaph3, and 6His-SNAP-IRSp53, pCS2 constructs were transfected into 293F cells by 293fectin reagent (Thermo Fisher Scientific) according to the manufacturer’s instructions 48 h before harvesting. pET and pGEX plasmids were transformed into BL21 pLysS *Escherichia coli* and induced overnight at 19°C. Cells were harvested, and pellets were resuspended in buffer containing 150 mM NaCl, and 20 mM Hepes, pH 7.4, with 2 mM 2-mercaptoethanol and EDTA-free cOmplete protease inhibitor tablets (Roche) before lysis by probe sonication. Following ultracentrifugation (40,000 rpm for 45 min in a 70Ti rotor), proteins were affinity purified on Ni-NTA agarose beads (Qiagen). Proteins were eluted from Ni-NTA beads by stepwise addition of increasing concentrations of 50–300 mM imidazole in a buffer containing 20 mM Hepes, pH 7.4, 150 mM NaCl, and 2 mM 2-mercaptoethanol. For all proteins apart from Diaph3, fractions were pooled and purified further using S200 gel filtration on an AKTA flow pressure liquid chromatography system (FPLC; GE Healthcare) in a buffer containing 150 mM NaCl, 20 mM Hepes, pH 7.4, and 5 mM DTT. Diaph3 was used directly after affinity purification without further steps to prevent loss of activity, which occurred within ∼9 h of purification. We were only able to tag Fascin and Diaph3 with eGFP, precluding examination of their correlation with each other.

To purify 6His-KCK-VASP, pET15b vector containing the 6His-KCK-VASP insert was transfected into Rosetta DE3 pLysS cells and induced overnight at 19°C. Cells were harvested, and pellets were resuspended in buffer containing 300 mM NaCl and 20 mM Hepes, pH 7.4, with 2 mM 2-mercaptoethanol and EDTA-free cOmplete protease inhibitor tablets (Roche) before lysis by probe sonication. Following ultracentrifugation (40,000 rpm for 45 min in a 70Ti rotor), proteins were affinity purified on cobalt agarose beads (Talon superflow; GE Healthcare). Proteins were eluted from cobalt beads by stepwise addition of increasing concentrations of 50–300 mM imidazole in a buffer containing 20 mM Hepes, pH 7.4, 300 mM NaCl, and 2 mM 2-mercaptoethanol. Fractions were pooled and concentrated using a 10,000 MWCO spin concentrator (Millipore). To purify 6His:SNAP:N-WASP/ZZ:WIP, the complex was expressed in 293F cells as described. The ZZ-WIP contains a TEV cleavage site between the tag and the protein. Lysates were incubated with IgG sepharose 6 beads (GE Healthcare) in XB buffer (100 mM KCl, 0.1 mM CaCl_2_, 1 mM MgCl_2_, and 10 mM Hepes, pH 7.4). Following extensive washing, Tobacco Etch Virus (TEV) protease was added in XB buffer containing 10 mM DTT and 10% glycerol and incubated overnight to cleave the N-WASP–WIP complex from the beads. The disposable column was drained to collect the cleaved protein complex and applied to a small volume of glutathione sepharose beads to sequester the TEV protease. For SDS-PAGE analysis, samples were boiled in SDS sample buffer, run on 4–20% polyacrylamide gels (Bio-Rad) according to manufacturer’s instructions, and analyzed by adding InstantBlue (Expedeon).

### Chemical labeling of proteins with fluorescent dyes

Labeling of SNAP-tagged recombinantly expressed proteins (6His-SNAP-Toca, 6His-SNAP-VASP, 6His-SNAP-Ena, 6His-SNAP–N-WASP/ZZ-WIP, and 6His-SNAP-IRSp53) was performed with 5–10 µM final protein concentration and 10 µM dye (SNAP-Surface Alexa Fluor488 [New England Biolabs; S9129S] or SNAP-Surface Alexa Fluor647 [New England Biolabs; S9136S], respectively) in 50 µl final volume with a buffer containing 150 mM NaCl, 20 mM Hepes, pH 7.4, 1 mM DTT, and 1% TWEEN 20. After incubation overnight at 4°C with protection from light and gentle rotation, excess dye was removed by two rounds of dialysis over 24 h using a 0.1-ml Side-A-Lyzer MINI Dialysis Device with a molecular cutoff of 20 kD (Thermo Fisher Scientific; 10237043) in a buffer containing 150 mM NaCl, 20 mM Hepes, pH 7.4, and 10% glycerol. Labeling of recombinantly expressed 6His-KCK-VASP was performed with ∼50 µM final protein concentration and 10–20-fold molar excess of maleimide dye (AF 568 C5 Maleimide [Life Technologies; A-20341] or AF 647 C5 Maleimide [Life Technologies; A-0347], respectively) in a 10-fold molar excess of tris(2-carboxyethyl)phosphine (TCEP). After incubation overnight at 4°C with protection from light and gentle rotation, the dye was removed by buffer exchange using a spin concentrator (Amicon Ultra-15 Centrifugal Filter Unit with an Ultracel-10 membrane (Merck Millipore; UFC901024) and a buffer containing 20 mM Hepes, pH 7.4, 300 mM NaCl, and 10% glycerol. Unlabeled skeletal rabbit muscle actin was prepared as previously described (Daste et al., 2017).

The muscle from the leg of a rabbit, killed the same day and kept on ice, was taken, and the fat and connective tissue was removed. The muscle was minced twice with a meat grinder and extracted in Guba Straub buffer (0.3 M NaCl, 0.1 M NaH_2_PO_4_ (2H_2_0), 5 mM Na_2_HPO_4_, 1 mM NaN_3_, 0.05 mM PMSF, 1 mM MgCl_2_, and 1 mM Na_4_P_2_O_7_, pH to 6.5, adding 1 ml 200 mM ATP just before use), stirring consistently for 10–15 min. The mixture was centrifuged at 3,000 rpm in J6-MC centrifuge (Beckman Coulter; rotor TY.JS 4.2), precooled to 4°C. The muscle residue was resuspended in 1 liter of buffer containing 4% NaHCO_3_ and 1 mM CaCl_2_ plus 9 liters distilled water (dH_2_0), stirred for 15 min, and filtered through four layers of cheesecloth. The muscle residue was diluted into 10 liters dH_2_0 and quickly squeezed through cheesecloth, working quickly as muscle swells at low ionic strength and F-actin is converted to G-actin and would be lost. The residue was suspended in 2.5 liters cold acetone and stirred at room temperature for 15 min and then filtered through cheesecloth. Acetone washing and filtering was repeated three or four times until the supernatant became clear. The muscle powder was spread out on filter paper and dried in the fume hood overnight. Once dry, it was stored at −80°C. Stringy parts of the muscle acetone powder were separated, and 5 g was weighed out and added to 100 ml ice-cold G-buffer (2 mM Tris, pH 8, 0.2 mM CaCl_2_, 0.5 mM NaN_3_, 0.2 mM Na_2_ATP, and 0.5 mM DTT). The mix was slowly stirred on ice for 30 min until viscosity increased. The suspension was carefully transferred into polycarbonate centrifuge tubes for 70Ti rotor (Beckman Coulter), topped up with G-buffer, and centrifuged at 20,000 *g* for 35 min at 4°C. The supernatant was filtered through two small pinches of glass wool packed into the neck of a funnel, then filtered with 0.45-µm followed by 0.22-µm filters. At room temperature, a final concentration of 0.8 M KCl and 2 mM MgCl_2_ was added. The actin was stirred slowly and consistently for 30 min at room temperature to let it polymerize, then stirred gently at 4°C to dissociate contaminating tropomyosin. The suspension was poured into tubes for the 70Ti rotor (Beckman Coulter) and centrifuged at 45,000 rpm (80,000 *g*) at 4°C to sediment the F-actin. 1–2 ml G-buffer was added, and the pellet was gently resuspended using a Teflon-coated rod. The actin was homogenized and transferred to a dialysis cassette. The actin was dialyzed in the cold room against 1 liter of G-buffer, then dialyzed again overnight. Dialysis continued by changing the G-buffer three times each over the following 2 d in the cold room. On day 4, the dialysis buffer was changed two or three times over 4 h in the morning. Then, the actin was centrifuged at 80,000 *g* for 2 h to sediment aggregates. An S200 gel filtration column was equilibrated with fresh G-buffer using an FPLC, and the G-actin was applied to the column and repeated as needed, avoiding the lower part of the tube containing the pellet of actin aggregates. The fractions were run on SDS-PAGE, and the pure actin-containing fractions were pooled and dialyzed into G-buffer minus NaN_3_. Sucrose was added to 5% final concentration, and 5-ml aliquots were snap frozen and then dried in a freeze dryer and stored at −80°C. Cold dH_2_0 was added to resuspend the actin, which could then be stored on ice for up to 1 mo.

### Quantitative Western blotting

A dilution series of high-speed supernatant *X. laevis* egg extracts as well as a dilution series of recombinant purified proteins were size separated by SDS-PAGE. Samples were transferred to a nitrocellulose membrane by wet transfer using a Bio-Rad Mini Trans-Blot Cell apparatus using transfer buffer (25 mM Tris, 192 mM glycine, 0.1% SDS, and 20% methanol) for 1 h at 0.38 A. Blots were rinsed with deionized water and blocked in Tris-buffered saline with 0.1% Tween 20 with 5% milk (skimmed, household milk powder) for 20 min at room temperature. Primary antibodies were diluted in 5 ml blocking buffer each and incubated with the membrane for 1 h at room temperature or overnight at 4°C. The membrane was incubated with IRDye 800CW Goat anti-Rabbit IgG Secondary Antibody (LI-COR Biosciences; catalog no. 926–32211) diluted in blocking buffer for 30 min at room temperature. Blots were imaged on a LI-COR BioSciences Odyssey CLx scanner using a solid-state diode laser at 785 nm. Intensities of the dilution series of the recombinant protein with known concentrations were measured using LI-COR image studio software and used as a standard curve for the extract samples. Blots were repeated at least twice for each protein. The TOCA-1 antibody (Ho et al., 2004), VASP antibody, and Fascin antibody (Lee et al., 2010) were raised in rabbits and were a kind gift from Marc Kirschner, Harvard University, Cambridge, MA. The N-WASP antibody was raised against full-length purified *X. tropicalis* 6His-SNAP–N-WASP/human ZZ-WIP in rabbit and affinity purified against the same protein sample. The Ena antibody was raised against full-length purified *X. laevis* 6His-SNAP-Ena in rabbit and affinity purified against the same protein sample.

### FLS assays

Supported lipid bilayers and high-speed supernatant frog egg extracts were made, and FLS assays were performed as previously described (Walrant et al., 2015). For snapshot (including FLS reinitiation and GFP-PLCδ-PH binding experiments), time-lapse, and immunolabeling data, a 1:6 dilution of 25 mg/ml egg extracts was used and optimized for FLS spacing and image segmentation by FLS Ace, with the addition of 10 µM unlabeled actin to give a final total actin concentration of 14 µM to ensure actin was not limiting for FLS length measurements. Typically, a final volume of 50 µl of reaction mix (2 mM DTT, 1× energy mix [50 mM phosphocreatine, 20 mM Mg-Adenosine triphosphate], 1 × XB buffer [100 mM KCl, 100 nM CaCl_2_, 1 mM MgCl_2_, 10 mM K-Hepes, pH 7.4, and 50 mM sucrose], 4.2 mg/ml high-speed supernatant *X. laevis* egg extracts, labeled proteins of interest, and 10 × XB to account for salt concentration) stored on ice was gently added to the supported lipid bilayer at room temperature. For FLS reinitiation experiments, FLSs were allowed to grow for 20 min with snapshot images collected over the next 5 min, after which the FLS mix was transferred to a well containing a fresh supported lipid bilayer. The transferred mix was allowed to incubate on the fresh membrane for a further 20 min, with snapshots then taken for the next 5 min. For the two protein combinations indicated in Table 1, actin was visualized using spinning disk confocal microscopy and the other proteins using HILO illumination on the same field of view. For the three protein combinations indicated in Table 2, Atto 390 labeled actin was present in all experiments and visualized using wide-field microscopy. The other proteins were visualized using HILO illumination on the same field of view. Together with the combinations in Table 1, all possible double combinations of proteins were performed with three repeats each, with the exception of GFP-Diaph3 and GFP-Fascin.

For high-rate velocity measurements, actin dynamics were monitored by addition of 250 nM GFP-utrophin–CH domain instead of labeled actin to prevent bleaching. For time-lapse videos, microscopic measurements were started 3 min after initiation. For snapshot images, microscopic measurements were performed at 20–30 min and for FRAP experiments 30–40 min after initiation. For FRAP experiments, a 1:4 dilution of egg extracts was used, with no additional actin and the same ratio of labeled protein, as it gave a larger tip complex, improving sensitivity.

### FLS immunofluorescence

FLS assays were performed for 20 min. To stabilize the actin bundle, unlabeled phalloidin (Thermo Fisher Scientific; P3457) was added to 66 nM concentration 15 min after initiation for 5 min. The FLSs were then washed twice with XB buffer containing 1× energy mix and 2 mM DTT. The structures were then fixed with 4% PFA in XB for 1 h. The fixative was quenched using 50 µM glycine in PBS for 15 min. FLSs were blocked with 10% goat serum in PBS for 30 min and then incubated with rabbit polyclonal antibodies (anti-Ena, anti-VASP, anti–N-WASP, and anti-Fascin) diluted 1/100 in block solution (10% goat serum in PBS) for 1 h. The FLSs were washed three times with PBS for 5 min. The structures were then incubated with fluorescent secondary antibody (Thermo Fisher Scientific; A21244) and fluorescently labeled phalloidin (Thermo Fisher Scientific; A12379) diluted 1/200 in block solution for 1 h. Finally, the FLSs were washed three times for 5 min with PBS. Assays were repeated at least three times for each antibody. VASP antibody and Fascin antibody (Lee et al., 2010) were a kind gift from Marc Kirschner, Harvard University, Boston, MA. The N-WASP antibody was raised against full-length purified *X. tropicalis* 6His-SNAP–N-WASP/human ZZ-WIP in rabbit and affinity purified against the same protein sample. The Ena antibody was raised against full-length purified *X. laevis* 6His-SNAP-Ena in rabbit and affinity purified against the same protein sample.

### Fluorescence microscopy

Microscopic images were acquired on a custom combined total internal reflection fluorescence/spinning disk confocal system supplied by Cairn Research based on a Nikon Eclipse Ti-E inverted microscope equipped with an iLas2 illuminator (Roper Scientific), CREST X-light Nipkow spinning disk, 250-µm NanoScanZ piezo driven z-stage/controller, and Lumencor Spectra X LED illumination using a 100× 1.49 NA oil immersion objective. Images were collected live at room temperature within the assay medium. A Photometrics Evolve Delta EM-CCD camera was used in 16-bit depth using Metamorph software version 7.8.2.0 (Molecular Devices). Atto-390 and Alexa Fluor (AF) 488, 568, and 647 samples were visualized using 460/50, 470/40, 560/25, and 628/40 excitation and 525/50, 585/50, and 700/75 emission filters, respectively. To monitor the simultaneous accumulation of different actin regulators to the FLS tip complex together with elongation of the actin bundle, we used highly inclined and laminated optical sheet illumination in up to three wavelengths and wide-field illumination (of Atto 390 actin) or spinning disk confocal optical sectioning in 3D (AF 647-actin, AF 560-actin, and AF 488-actin). We employed rapid sequential time-lapse imaging of actin and other proteins in different combinations to visualize bundle dynamics (Table 1 and Table 2). For fluorescence recovery after photobleaching experiments, a reference HILO image of the protein of interest and a confocal z-stack of the actin channel were acquired. A maximum of eight circular regions of interest (ROIs) of 20 × 20 pixels (2.97 × 2.97 µm; e.g., four FLS tips and four corresponding background regions) were selected. Five frames were recorded in 1-s intervals before bleaching using the 488-nm or 561-nm laser at 25% power for two iterations. Fluorescence recovery was recorded by the corresponding laser line at 5% laser power for 120 frames, with intervals of 0.5 s for the first 40 frames and 1 s for all following frames. Changes in fluorescence intensity in the ROI after photobleaching were analyzed in ImageJ using a publicly available script developed at the Image Processing School Pilsen 2009 according to procedures previously described (Bancaud et al., 2010). The bleach frame was identified by the largest frame-to-frame drop in intensity. For each analysis, four ROIs—bleached FLS, nonbleached FLS, bleached background, and nonbleached background—were specified. The nonbleached regions were selected in close proximity to the corresponding bleached regions to account for local background. First, a background ROI intensity subtraction from the measured values was performed on the raw data, and the prebleach frames were normalized to 1. Loss of fluorescence during postbleach acquisition was corrected for based on the unbleached ROI; furthermore, the bleach frame was normalized to 0.

### FLS image analysis

For extracting data from spinning disk confocal and HILO microscopy images of FLSs, we developed FLSAce, a plugin for ImageJ/Fiji (Schindelin et al., 2012) that maps actin structures in z-stacks and measures signal intensity in images representing other channels. FLSs are segmented using a 2D Difference of Gaussians filter to define positions independently in each XY plane, which are then traced through z starting from each position found in the base plane using a greedy algorithm. The plugin has a user-friendly interface allowing the base plane, DoG σ and k values, thresholding method, minimum required length for traced FLSs, and the maximum tracing radius to be set and tested on a single actin stack to ensure accurate FLS detection. These parameters can then be used in batch mode to map FLSs in many actin stacks taken at a series of time points and to measure as many associated images as required. Linear assignment between structures detected in multiple time points allows for phenotypic analysis of individual FLSs throughout their lifetime. Batch mode is parallelized for speed, and an XML configuration file is used to define gene names, regular expressions to determine which images correspond to each gene, and the segmentation parameters to use for each. The actin-based segmentation is used to measure signal intensity in FLS bases in any additional (total internal reflection fluorescence) channel. To alleviate systematic spatial shifts stemming, for example, from chromatic aberration, we performed translation image registration using the actin base slice as a reference. Local background in each channel is determined by averaging the fluorescence intensity in a ring two to three times the size of the FLS base, excluding any neighboring FLS bases. To efficiently process our GFP-utrophin–CH-based high time-resolution time lapse videos, we reimplemented the above algorithm in Python using algorithms from scikit-images.

### Postprocessing of snapshot data

We filtered out segmented FLSs with an effective diameter of <0.5 µm because base size information as well as fluorescent intensity measurements become unreliable at or below the resolution limit. When considering FLS length distributions, we truncated the distribution to lengths between 5 µm and 20 µm to exclude structures that have not yet matured into FLSs and artifacts stemming from the lower z-resolution in our confocal stacks. Fluorescence intensities of observed tagged proteins were determined by subtracting the local background intensity from the intensity averaged over all pixels in the FLS base area. To determine protein–protein and protein–morphology correlations, we calculated Spearman correlation values for each field of view separately and averaged over the resulting ensemble. Unless indicated otherwise, fluorescence intensities of actin regulatory proteins were measured at the membrane/tip complex plane.

### Postprocessing of time-lapse data

Artificial breaks in trajectories can occur due to segmentation errors. We took care to repair these breaks by merging trajectories with average base positions closer than 1 µm, within six time points and no temporal overlap, using a greedy algorithm. To reduce noise from image segmentation errors, we smoothed FLS length and growth velocity trajectories using a Savitzky-Golay filter of order 3 with a window of 11 time points.

### *Drosophila* stocks

Flies were raised and crossed at room temperature. The wild-type strain used was *white[1118]*. Fascin mutant *sn[28]* and the transgene *UAS-GFP-fascin* (kindly provided by Brian Stramer, Kings College London, London, UK) are described in FlyBase. *Mef2-Gal4* was used to drive in myotubes, *Btl-Gal4* in trachea, and *engrailed-Gal4* in the leading edge cells in dorsal closure. To make *GFP-fascin; en-Gal4 UAS-cd8mCherry / (CyO)* flies, the original flies used for knock-in were *y[1] sc[1] v[1];; {y[+t7.7] v[+t1.8]=nanos-Cas9}attp2.* Fascin was tagged by CRISPR/Cas9–mediated genome editing with GFP at the N-terminus. GFP insertion was before the first amino acid of Fascin, with the addition of a linker sequence such that the fusion protein junction corresponds to TKASSSSM. Two gRNA sites were chosen, one at the GFP insertion point and the other 1.4 kb upstream in the 5′ UTR.

For donor plasmid, pTv-[w^+^] *fascin* GFP was constructed by In-Fusion cloning of PCR generated 5′ and 3′ homologous arms, GFP, and ClaI/XhoI cut pTv-[w^+^] vector. 1.4-kb 5′ homologous arm amplified from CFD2 genomic DNA with primers 5′-TAT​TCG​AAT​CTG​CAG​CGT​TTA​GCG​TTA​CTG​ACT​GTG​GGC-3′ and 5′-CTT​TAC​TCA​TGG​TGC​TGA​TGG​GAG​CAA​TCT-3′. 1.3-kb 3′ homologous arm was amplified from CFD2 genomic DNA with primers 5′-TTC​GAG​TTC​ATC​TAT​GAA​CGG​CCA​GGG​CTG​CGA-3′ and 5′-AAT​GGC​ACT​GTT​ATC​GCT​ATC​ATC​TAT​TGA​GCC​ATT​TAG​CCA-3′. The gRNA sequence is split by the insertion of GFP, so it was not necessary to introduce silent mutations to prevent cleavage of the donor plasmid. GFP was amplified with primers 5′-AGC​ACC​ATG​AGT​AAA​GGA​GAA​GAA​C-3′ and 5′-TTC​ATA​GAT​GAA​CTC​GAA​GCT​TTG​TAT​AGT​TCA​TC-3′.

For gRNA plasmid, Fascin gRNA target sequences were cloned into pCFD4-U6:1_U6:3tandemgRNAs plasmid (a gift from Simon Bullock, MRC Laboratory of Molecular Biology, Cambridge, UK); Addgene plasmid #49411) by In-Fusion cloning (Port et al., 2014). Primers 5′-TAT​ATA​GGA​AAG​ATA​TCC​GGG​TGA​ACT​TCG​CTC​CCA​TCA​GCA​CCA​TGA​AGT​TTT​AGA​GCT​AGA​AAT​AGC​AAG-3′ and 5′-ATT​TTA​ACT​TGC​TAT​TTC​TAG​CTC​TAA​AAC​GCT​CGC​AGC​CCT​GGC​CGT​TCC​GAC​GTT​AAA​TTG​AAA​ATA​GGT​C-3′ were used to amplify and introduce the two Fascin gRNAs from pCFD4-U6:1_U6:3tandemgRNAs, which were then cloned into BbsI cut pCFD4-U6:1_U6:3tandemgRNAs.

CDF2 nos-Cas9 fly embryos (genotype: y1 P(nos-cas9, w^+^) M(3xP3-RFP.attP)ZH-2A w*; a gift from Simon Bullock) were injected with 250 ng/µl each of donor and gRNA constructs. Male flies were crossed with C(1)DX balancer.

To make *Scar/WAVE-NeonGreen; en-Gal4 / CyO; UAS-cd8mCherry / TM2TM6* flies, SCAR/WAVE was tagged by CRISPR-mediated genome editing with mNeonGreen at the C-terminus. mNeonGreen insertion was between the final amino acid of Scar/WAVE and the stop codon, with the addition of a linker sequence such that the fusion protein junction corresponds to TSASSSSM. Two gRNA sites were chosen, which flanked the insertion site, with the PAM motifs being separated by six nucleotides.

For donor plasmid, pTv-[w^+^] *Scar/WAVE* mNeonGreen constructed by In-Fusion cloning of PCR generated 5′ and 3′ homologous arms, mNeonGreen, and HindIII/XhoI cut pTv-[w^+^] vector. 1.2-kb 5′ homologous arm amplified from CFD2 genomic DNA with forward primer 5′-GAA​TCT​GCA​GCT​CGA​TCA​ACG​GCT​CTA​ATA​TCT​CAC​ATT​C-3′ and reverse primer 5′-CGA​TGA​GCT​CGA​AGC​TGA​AGT​TTC​GTT​CGG​TTC​CAT​CCA​GCC​CTC​GC-3′. The reverse primer has two silent changes from genomic sequence in the gRNA target sequence (C to T and T to A at positions 16 and 19) to prevent cleavage of the donor plasmid. 1.4-kb 3′ homologous arm amplified from CFD2 genomic DNA with forward primer 5′-CAA​GTG​ATC​CCT​GAT​AAA​TTC​GTT​AAA​GCC​TG-3′ (with silent changes from genomic sequence of T to A and C to T at positions 16 and 19) and reverse primer 5′-TCG​AAA​GCC​GAA​GCT​GCC​CAC​CGC​AAT​TAG​CTT​ATA​TTG-3′. mNeonGreen was amplified from plasmid pNCS mNeonGreen (Allele Biotech) with primers 5′-GCT​TCG​AGC​TCA​TCG​ATG​GTG​AGC​AAG​GGC​GAG​GAG​GAT​AAC​ATG​GCC​TC-3′ and 5′-ATC​AGG​GAT​CAC​TTG​TAC​AGC​TCG​TCC​ATG​CCC​ATC-3′.

For gRNA plasmid, Scar/WAVE gRNA target sequences were cloned into pCFD4-U6:1_U6:3tandemgRNAs plasmid by In-Fusion cloning. Primers 5′-TCC​GGG​TGA​ACT​TCG​GAT​GGA​ACC​GAA​CGA​AAC​ATG​TTT​TAG​AGC​TAG​AAA​TAG​CAA​G-3′ and 5′-TTC​TAG​CTC​TAA​AAC​TGA​TTA​ACT​CGT​TAA​AGC​CTC​GAC​GTT​AAA​TTG​AAA​ATA​GGT​C-3′ were used to amplify and introduce the two Scar/WAVE gRNAs from pCFD4-U6:1_U6:3tandemgRNAs, which was then cloned into BbsI cut pCFD4-U6:1_U6:3tandemgRNAs.

CDF2 nos-Cas9 fly embryos were injected with 250 ng/µl each of donor and gRNA constructs. Two independent lines were established with mNeonGreen-tagged Scar/WAVE: 15 X F0 male flies were crossed with Gla/CyO balancer (injected males were prescreened by PCR with the forward 5′ homologous arm primer and a reverse mNeonGreen primer 5′-CAC​CAT​GTC​AAA​GTC​C-3′). 4× Positive F1 flies (prescreened as for F0 flies) were then crossed with Gla/CyO balancer line. Correct integration of mNeonGreen was confirmed in two lines by PCR and sequencing across the entire donor sequence. PCR was also performed with primers flanking the donor sequence to confirm the size of the integrated fragment.

To make *GFP-ena[w*^*+*^*]/CyO ; UAS-cd8mCherry* flies, *ena* was tagged with GFP in its endogenous gene locus using genomic engineering (Huang et al., 2009). After deletion of almost the entire *ena* ORF and 3′ UTR (2R: 19,158,510–19,163,715; release r6.07), including the N-terminal EVH1 domain by homologous recombination using a 5.3-kb 5′ and a 3.4-kb 3′ homology arm, it was replaced with an attP integration site (Ena knock-in platform: Ena[GX]). A wild-type *ena* genomic rescue construct created by subcloning a 5,203-bp fragment from the Bac RP98-01N09 (Berkeley Drosophila Genome Project) was reinserted, containing 224 bp upstream of the nucleotide corresponding to the first nucleotide of the Ena EVH1 domain, and 2,025 bp downstream of the stop codon of *ena*, into the attB vector pGE-attB-GMR (Huang et al., 2009). The GFP (variant mGFP6) coding sequence was inserted at the N-terminus of the EVH1 domain with a short linker amino acid sequence at the C-terminus of GFP, the fusion protein junction that corresponds to YKASSSSEQS. GFP will be fused to all annotated isoforms of Ena.

### Live imaging and data analysis of *Drosophila* embryo filopodia

Dechorionated embryos (washed in 50% bleach) were mounted on a glass-bottomed dish with heptane glue and submerged in water. To identify GFP-Ena homozygous embryos, balancer chromosome CyO with Dfd-YFP was used, and we did negative selections for Dfd-YFP expression in the head region of embryos. Live microscopy was performed on an inverted Leica TCS-SP5 equipped with a 63× 1.4 NA Plan Apo oil immersion objective at room temperature. To visualize filopodial movement and respective protein intensities within filopodia in dorsal closure, embryos were imaged at the end of stage 14. Microscopic measurements were performed by taking z-stacks of 7–11 z sections (0.5-µm spacing). Time lapses were taken at 15-s intervals for 10 min. Ena and Scar/WAVE intensities at the tips of filopodia were quantified manually using ImageJ. Filopodial lengths and fascin intensities were quantified using our previously developed open-source pipeline Filopodyan (Urbančič et al., 2017). Using ImageJ, a maximum projection of the respective stacks was applied for filopodia reconstruction. A combination of automated detection with some manual editing was used to track identified filopodia over time.

### Tracing and tracking of filopodia in *Drosophila* myotube time-lapse movies

Time-lapse stacks of myotubes in *Drosophila* early and late stage-16 embryos from the dataset used in Richier et al. (2018) were reanalyzed using the following machine-learning pipeline: (1) Preprocessing: Stacks were z-projected to single images using average pixel intensities. The resulting images were preprocessed by applying a local thresholding filter (from the scikit-image library; Pedregosa et al., 2011) with a size of 11 pixels and a Gaussian profile. (2) Training set: Manually traced filopodia from 25 time-lapse movies (see Richier et al., 2018 for details) were used as a training set. The 13 × 13 pixel neighborhood of every pixel belonging to a filopodium was extracted and labeled as filopodia positive. Pixel neighborhoods of the same size from randomly chosen pixels not belonging to a filopodium were extracted and labeled as filopodia negative. The number of negative labels was set to 20× the number of positive labels. (3) Classification: A random forest classifier (Ho, 1995) from the scikit-learn library (Pedregosa et al., 2011) with a maximum depth of 50 and a minimum number of five samples per leaf was trained on the set of positively and negatively labeled pixel neighborhoods. This random forest classifier was then used to label pixels from not-annotated images, resulting in a binary mask where a value of zero means background and one indicates that a pixel is part of a filopodium. (4) Cleanup: To minimize spurious detections, connected regions of filopodia-labeled pixels containing <40 pixels were removed. (5) Clustering: To identify clusters of pixels belonging to the same filopodium, the DBSCAN algorithm (Ester et al. 1996. Proceedings of the Second International Conference on Knowledge Discovery and Data Mining.) from the scikit-learn library (Pedregosa et al., 2011) with a neighborhood search size of 7 pixels and a minimum number of points of 20 was used. (6) Skeletonization and joining: The resulting patches of pixels representing filopodia were reduced to thin lines via the skeletonization algorithm (Zhang and Suen, 1984) from the scikit-image library (van der Walt et al., 2014). Due to weak signal and obscuring features, filopodia might be split into two or more fragments. To join these, fragment tips were connected via a straight line if the two tips were within a 20-pixel distance and if the vectors formed by averaging the local tangents of the three pixels closest to both tips formed an angle no larger than 30°. (7) Measurement of filopodial lengths: The length of filopodia was measured by adding the distances between a pixel and its closest neighbor iteratively starting at one end. When branch points were detected, the branches were connected such that the resulting filopodium would be as long as possible. The remaining branches were split off as separate filopodia. (8) Frame linking: Filopodia were tracked across frames using the Hungarian linear assignment algorithm (Kuhn, 1955) from the scipy library (http://www.scipy.org/) with the following cost function for each possible match of filopodia between frame and frame *n* + 1: $C=0.6\times \sqrt{(x_{1}-x_{2}{)}^{2}+(y_{1}-y_{2}{)}^{2}}+0.4\times (L_{1}-L_{2}),$ where *x*_1_, *x*_2_, *y*_1_, and *y*_2_ refer to the center-of-mass coordinates and *L*1 and *L*2 to the lengths of the two filopodia in question. Additionally, filopodia whose center of mass was more than 30 pixels apart between frames were not allowed to link. (9) Growth velocity: Filopodia that persisted for less than five frames were removed. The remaining filopodial length trajectories were smoothened, and their derivative (the growth velocity) was extracted using the Savitzky-Golay filter (Savitzky and Golay, 1964) from the scipy library (http://www.scipy.org/) with a window length of 5 and a polynomial order of 2.

### Mathematical analysis

Like in the overdamped limit of Langevin equations (i.e., low Reynolds number), the velocity can simply be set equal to the force. We then assume that the force (and thus the growth velocity) acting on the growing FLS *F* is a function of the fluctuating concentrations of a set of proteins *A_i_* with *i* = 1,...,*K*. We assume that the proteins obey independent Ornstein-Uhlenbeck relaxation processes:

$$\;\;dA_{i}=\theta ({\tilde{A}}_{i}-A_{i})dt+dW(t);$$ (1)

that is, the protein concentrations fluctuate around a baseline concentration ${\tilde{A}}_{i}.$ The parameter θ>0 determines the relaxation time scale of fluctuations. The Brownian process W(t) has zero mean and obeys the correlations $\langle W(t)W(s)\rangle =2\theta \eta^{2}\delta (t-s)$ with the noise controlled by the parameter η. We take the difference between concentration and baseline as $X_{i}=A_{i}-{\tilde{A}}_{i}$; hence, the $X_{i}$ are normally distributed with zero mean in the long time limit.

One thus expects force to arise from a combination of protein concentration, as indicated by the fact that we see weak but nonzero correlations between FLS lengths and many proteins. The most minimalistic and generic version of such a theory is to assume that each type of protein is independent of every other type of protein (uncorrelated random variables) but that they act on force in either an additive or a multiplicative way. For instance, if two proteins $X_{1}$ and $X_{2}$ act on the force via a common given complex, the output would be expected to be multiplicative ($F=X_{1}X_{2}$), while if they interact in an independent pathway, the output would be expected to be additive ($F=X_{1}+X_{2}$). In general, we can thus see the output force as a sum of M products of N fluctuating protein concentrations:

$$F^{\left(M,N\right)}=\sum_{i=1}^{M}\prod_{j=1}^{N}X_{N\left(i-1\right)+j}.$$ (2)

Strikingly, an exact Laplacian force distribution can be achieved by the combination:

$$F^{\left(2,2\right)}=X_{1}X_{2}+X_{3}X_{4}.$$

This can be shown analytically. Indeed the product of two standard normal distributions $Z=X_{1}X_{2}$ is distributed according to a modified Bessel function of the second kind:

$$P(Z=z)=\frac{1}{\pi }\int_{0}^{\infty }\frac{1}{\left|x\right|}e^{-(x^{2}+z^{2}/x^{2})/2}dx=\frac{1}{\pi }K_{0}(\left|z\right|).$$

The sum of two products $W=Z_{1}+Z_{2}$ is then distributed according to a Laplace distribution:

$$P(W=w)=\frac{1}{\pi^{2}}\int_{-\infty }^{\infty }K_{0}(\left|z\right|)K_{0}(\left|z-w\right|)dz\;\;\;\;\;\;\;\;\;\;\;\;\;\;\;\;\;\;=\sqrt{\frac{w}{2\pi }}K_{1/2}(\left|w\right|)=e^{-\left|w\right|}.$$

To simulate FLS growth, we generated K=MN artificial protein concentration traces by integrating Eq. 1 using the Euler-Maruyama method with a time step of 1 s and combined them according to Eq. 2 to generate the force. We then arrived at FLS length trajectories L(t) by temporal integration of the equation

$$\frac{dL}{dt}=F,$$

with the conditions L(t)≥0 and L(t=0)=0.

### Biochemical connection

To make the connection between fluctuating protein concentrations and instantaneous force/growth velocity more tangible, we present a chemical reaction network consisting of 2*M* species *A_i_*, which interacts in pairs to form *M* complexes *B_j_*. The complexes are subject to decay. The reaction network is thus:

$$O\overset{\sigma }{\to }A_{i},i=0,\ldots ,2M-1,$$

$$A_{2j}+A_{2j+1}\overset{\rho }{\to }B_{j},j=0,\ldots ,M-1,$$

and

$$B_{j}\overset{\eta }{\to }O,j=0,\ldots ,M-1.$$

The mass action rate equations for the single species are then given by

$${\dot{a}}_{2j}=\sigma -\rho a_{2j}a_{2j+1}$$

and

$${\dot{a}}_{2j+1}=\sigma -\rho a_{2j}a_{2j+1},$$

with the (degenerate) steady-state solutions $a_{i}=\sqrt{\sigma /\rho }$. Therefore, we can write these concentrations in terms of the deviations (fluctuations) around the steady state:

$$\delta a_{i}=a_{i}-\sqrt{\frac{\sigma }{\rho }}.$$

Note that we are using the rate equations here even though the system of interest is far from equilibrium and mass action kinetics do not strictly apply. However, since we are interested in fluctuations around an average, we used them as a starting point. The rate equations for the complexes, expressed in terms of fluctuations, are given by

$$\begin{array}{l} {\dot{b}}_{j}=\rho a_{2j}a_{2j+1}-\eta b_{j}=\\ \sigma +\rho \delta a_{2j}\delta a_{2j+1}+\sqrt{\sigma \rho }(\delta a_{2j}+\delta a_{2j+1})-\eta b_{j}, \end{array}$$

with the total velocity for an FLS reading:

$$\nu \;\propto \sum_{j=0}^{M-1}{\dot{b}}_{j}.$$

Thus, there are three contributions to the steady-state concentrations of the complex $b_{j}$ (and conversely to FLS speed). The first contribution arises from the steady rate of production σ, which dictates the average complex concentration. The product of such terms gives rise to a constant nonzero velocity of FLS growth. However, at steady state, FLS net average velocity is close to zero because of depolymerization processes we have not modeled so far. Monomer availability, for instance, is a straightforward way to couple net polymerization and net depolymerization, ensuring that this first nonzero average contribution drops out. The second contribution $\delta a_{2j}\delta a_{2j+1}$ has already been discussed in the manuscript and involves the product of Gaussian fluctuations, yielding generically exponential tails for *v* (and in the limit of two sums, an exact Laplace distribution). The third contribution $\sqrt{\sigma \rho }\delta a_{2j}$ is a cross-term that involves the average concentration of one of the species and the fluctuation of the others and is thus Gaussian distributed. However, our data are consistent with species $a_{i}$ with large variance compared with the mean. In this limit, the third contribution becomes negligible, and the global distribution for velocities approaches a Laplace distribution rapidly, with the distribution in effect controlled by ${\dot{b}}_{j}\approx \rho \delta a_{2j}\delta a_{2j+1}$. One should note that in this limit of large variance, the Ornstein-Uhlenbeck equations for the concentrations $a_{i}$ must be amended with reflective boundary conditions at zero to avoid negative concentrations (or alternatively, a steep potential around 0). Importantly, we tested that this still generically yielded Laplace-like distributions, for instance, when combining regulators of growth versus regulators of shrinkage, effectively generalizing the expression above to

$$F=\sum_{i=1}^{M}(-1{)}^{i}\prod_{j=1}^{N}X_{N(i-1)+j},$$

with each species $X_{N(i-1)+j}$ strictly positive and obeying the modified stochastic dynamics mentioned above (as mentioned above, we assume that there are feedback mechanisms to ensure that the sum of all averages is zero, yielding steady-state conditions for the force/velocity).

Finally, under the assumption that force/growth velocity generation is directly dependent on the concentrations of each of the regulatory complexes, we arrived at the sum of products rule mentioned in the main text:

$$\nu \;\propto \sum_{j=0}^{M-1}{\dot{b}}_{j}\approx \sum_{j=0}^{M-1}\rho \delta a_{2j}\delta a_{2j+1}.$$

### Data availability

All data are available in the main text or the supplementary information. All data, code, and materials used in the analysis are available for the purposes of reproducing or extending the analysis. mNeonGreen constructs are supplied by the Allele Biotech repository.

### Online supplemental material

Fig. S1 shows the concentration quantification of our labeled proteins in egg extracts, together with the purified protein gels. Fig. S2 contains the number and morphology of FLSs over time, showing that extracts are not overly depleted of protein after an assay and that FLSs grown from previously used extract have similar lengths and counts. Fig. S3 shows FLS numbers for the correlation matrix shown in Fig. 4 B and similar matrices for large and small FLS width cutoffs. Fig. S4 displays an analysis of protein intensities along the FLS shaft. Fig. S5 shows length distributions for a number of double combinations of proteins. The Mathematical analysis section includes the derivation of the sum-of-products rule mentioned in the text and thereby how a Laplace-shaped growth velocity distribution can arise from fluctuating regulatory proteins. Video 1, Video 2, Video 3, Video 4, Video 5, and Video 6 show examples of filopodia in *Drosophila* embryos during dorsal closure, tracheal cell growth, or myotube growth, where either Ena or Scar was labeled. Video 7 shows a side view of growing FLSs. Video 8 shows Fascin in filopodia in *Drosophila* embryos undergoing dorsal closure. A supplemental data Excel file contains 21 tabs that display the data used to generate all the graphs shown in the figures as follows: Dataset 1 contains the filopodia length and protein intensity data points for Fig. 1, J, K, and L. Dataset 2 contains the filopodia length data from wild-type, Ena- and Scar-tagged *Drosophila* dorsal closure cells for Fig. 1 M. Dataset 3 contains the averaged protein intensity time series data shown in Fig. 3, A and B. Dataset 4 contains the number of local maxima shown in Figs. 3, D and E. Dataset 5 contains FLS morphology and protein intensity data used to generate Fig. 4, B, D–F, Fig. S3, A–C, Fig. S4, A–F, and Fig. S5. Dataset 6 contains the FLS morphology and protein intensity data from immunostaining experiments used to generate Fig. 4 C. Dataset 7 contains the FLS length and velocity time series shown in Fig. 5 A. Dataset 8 contains the FLS velocity data used to generate Fig. 5, B, F, and G. Dataset 9 contains the FLS length data used to generate Fig. 5, D and E. Dataset 10 contains the FLS growth persistence time data shown in Fig. 5 E. Dataset 11 contains the FLS FRAP time series data used to generate Fig. 6, A and B. Dataset 12 contains the FLS growth velocity and protein intensity data used to generate Fig. 6 D. Dataset 13 contains the fascin intensity and filopodia length data used to generate Fig. 7, B and D. Dataset 14 contains the fascin intensity in FLS data shown in Fig. 7 C. Dataset 15 contains the filopodia length data in fascin mutants shown in Fig. 7, E and F. Dataset 16 contains the *Drosophila* myotube filopodia growth velocity data used to generate Fig. 7, G and H. Dataset 17 contains the measured protein concentrations in HSS egg extracts shown in Fig. S1 B. Dataset 18 contains the FLS rate of appearance as a function of time shown in Fig. S2 A. Dataset 19 contains the FLS tip complex area and FLS length as a function of time shown in Fig. S2, B and C. Dataset 20 contains the measured protein intensities in extracts after exposure to glass, bilayer, and FLS conditions shown in Fig. S2, D. Dataset 21 contains the FLS length and count before and after restarting an assay with an already-used extract shown in Fig. S2, E and F.

## Supplementary Material

Data S1 displays all data used to generate all the graphs shown in the figures.
